# Natural Flavonoids Genistein and Baicalein as Well-Tolerated Radiosensitizers to Enhance the Efficacy of ^177^Lu-PSMA617 in Prostate Cancer: In Vitro and In Vivo Studies

**DOI:** 10.34133/research.1314

**Published:** 2026-06-10

**Authors:** Congjie He, Yuting Shao, Ying Bao, Jicong Li, Hongwei Si, Jian He

**Affiliations:** ^1^ Department of Nuclear Medicine, Nanjing Drum Tower Hospital Clinical College of Nanjing University of Chinese Medicine, Nanjing, China.; ^2^ Beijing Atomic Energy Institute, Beijing, China.; ^3^ Department of Nuclear Medicine, The First Affiliated Hospital of Anhui Medical University, Hefei, China.; ^4^ Department of Nuclear Medicine, Nanjing Drum Tower Hospital, Nanjing, China.

## Abstract

The efficacy of prostate-specific membrane antigen (PSMA)-targeted radioligand therapy in metastatic castration-resistant prostate cancer is limited by tumor heterogeneity and intrinsic radioresistance. This study investigates 2 natural flavonoids, genistein and baicalein, as potential radiosensitizers to improve the therapeutic effectiveness of ^177^Lu-PSMA617 therapy. In vitro, combined treatment with low-toxicity concentrations (5 to 15 μM), based on cellular median inhibitory concentration (IC_50_) of either flavonoid, dose-dependently enhanced the cytotoxicity of ^177^Lu-PSMA617 against the human prostate cancer cell line LNCaP, reducing its IC_50_ to 14.2% to 45.1% of that with monotherapy. All combination regimens demonstrated synergistic interaction, with combination indices below 0.82. Mechanistically, the combinations (particularly at 10 μM) induced a pro-oxidant shift (increasing reactive oxygen species by 78% to 144%), amplified DNA damage (γ-H2AX increased by 85% to 115%), and promoted apoptosis (caspase-3 activity increased by 325% to 389%) while suppressing prosurvival p-AKT. Transcriptomic profiling further revealed that both flavonoids induced a pro-apoptotic gene signature and markedly modulated multiple cancer-related pathways, particularly inhibiting key DNA double-strand break repair pathways such as nonhomologous end-joining. In vivo, combination therapies profoundly inhibited tumor growth (50.2% to 78.1% reduction versus monotherapy) and extended survival, with no overt systemic toxicity observed in this preliminary assessment. This work establishes genistein and baicalein as effective, multi-targeted radiosensitizers that enhance ^177^Lu-PSMA617 efficacy by cooperatively amplifying DNA damage, inhibiting repair mechanisms, and activating the apoptotic cascade. Their natural origin and established safety profile underscore their translational potential. Thus, genistein and baicalein represent promising novel adjuvants for prostate cancer radioligand therapy.

## Introduction

Prostate cancer remains a leading cause of cancer-related morbidity and mortality among men globally, with its incidence rising steadily in many regions, including China [1,2]. While localized prostate cancer can achieve favorable outcomes through surgery or radiotherapy, a substantial proportion of patients progress to metastatic castration-resistant prostate cancer (mCRPC). mCRPC represents an aggressive, highly heterogeneous disease stage characterized by progression despite androgen deprivation therapy (ADT). It carries a poor prognosis, with limited treatment options often accompanied by resistance, and a median survival typically under 20 months [3], rendering it the primary cause of cancer-associated mortality among men with prostate cancer.

The advent of prostate-specific membrane antigen (PSMA)-targeted radioligand therapy (RLT), notably ^177^Lu-PSMA617, has transformed the therapeutic landscape for PSMA-positive mCRPC. By delivering β radiation directly to PSMA-expressing tumor cells, ^177^Lu-PSMA617 induces lethal DNA damage and has demonstrated markedly survival benefits in pivotal trials [4,5]. However, its clinical effectiveness is constrained by primary and acquired resistance mechanisms, including heterogeneous or suboptimal PSMA expression, robust DNA damage repair (DDR) pathways, and the activation of prosurvival signaling [6,7]. While resistance to RLT is multifactorial, involving elements such as tumor hypoxia, cancer stem cells, and cell cycle redistribution [8–10], this study specifically focuses on the modulation of DDR and prosurvival signaling as a primary strategy to enhance radiosensitivity. Such constraints highlight the critical necessity for rationally designed combination approaches, aimed at enhancing tumor radiosensitivity and optimizing treatment efficacy.

Current clinical approaches to augment PSMA-RLT involve combinations with chemotherapy, next-generation androgen receptor pathway inhibitors (e.g., enzalutamide), or poly(adenosine diphosphate-ribose) polymerase inhibitors (PARPis; e.g., olaparib) [11,12]. Although promising, these strategies can introduce overlapping toxicities, necessitate specific molecular alterations for optimal efficacy (e.g., HRR mutations for PARPi), and contribute to the escalating economic burden of oncology care [13]. Consequently, there is growing interest in naturally derived, multi-targeted compounds, which could serve as promising radiosensitizers due to their improved therapeutic index and cost-effectiveness [14].

Among these, the flavonoids genistein (a soy isoflavone) and baicalein (from *Scutellaria baicalensis*) are compelling candidates. Both compounds have demonstrated the ability to modulate pathways relevant to radioresistance. Genistein can inhibit DDR proteins (e.g., DNA-PKcs) and survival signaling, while baicalein exhibits pro-oxidant activity and modulates oncogenic pathways such as phosphatidylinositol 3-kinase (PI3K)/AKT [15–17]. Crucially, these compounds have a history of dietary use and are associated with low systemic toxicity in preclinical and human studies, presenting a distinct safety advantage for combination regimens [18]. Their potential to act as cost-effective oral adjuvants could address substantial economic and logistical challenges in modern cancer care.

Despite their individual promise, whether these flavonoids can specifically overcome the acquired and intrinsic resistance to ^177^Lu-PSMA617—the most pressing clinical obstacle in PSMA-RLT—has never been investigated. We hypothesized that these flavonoids would act as effective and safe radiosensitizers to directly address this resistance. This study aims to (a) evaluate the synergistic efficacy of genistein and baicalein with ^177^Lu-PSMA617, (b) dissect the underlying molecular mechanisms, and (c) assess the in vivo therapeutic efficacy and safety of these combinations. Our findings provide a robust rationale for repurposing these natural compounds as a novel, mechanism-based adjuvant strategy specifically designed to counteract intrinsic radioresistance to PSMA-targeted RLT in mCRPC.

## Results

### Transcriptomic profiling reveals differential molecular responses to flavonoids

**Fig. 1.**
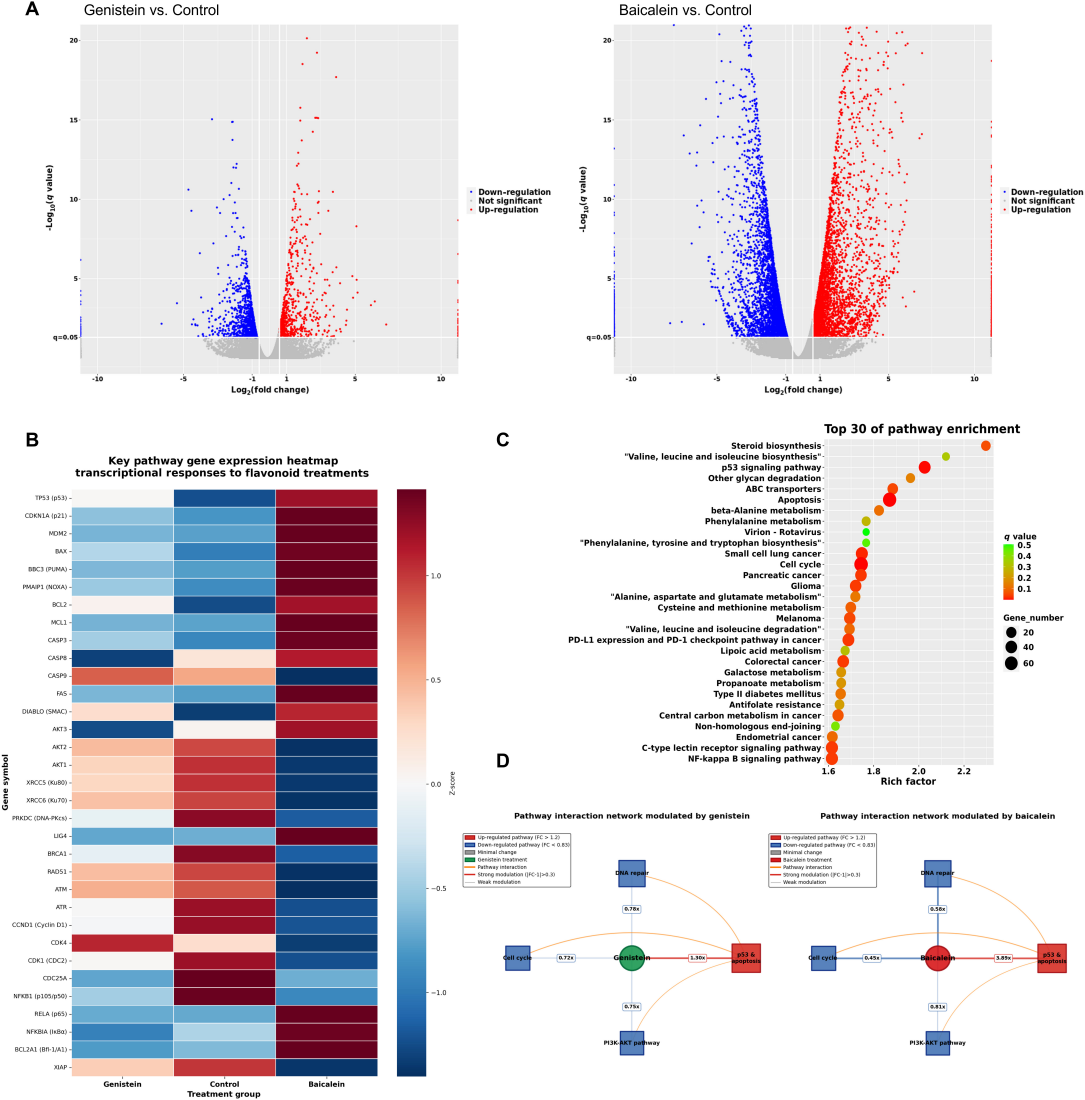
Transcriptomic profiling reveals differential responses to flavonoids in LNCaP cells. (A) Volcano plots of DEGs after genistein (left) or baicalein (right) treatment (20 μM, 24 h). Baicalein elicited stronger fold changes; genistein’s effects were broader but milder. (B) Heatmap of key genes across 4 functional modules. Baicalein induced coherent pro-apoptotic/anti-repair reprogramming; genistein’s changes were more subtle. (C) KEGG pathway enrichment for baicalein (top 8 significant terms). (D) Pathway-interaction networks modulated by genistein (left) and baicalein (right). Node color indicates regulation direction/magnitude; edge thickness represents modulation strength. Values denote average fold change per pathway.

RNA-sequencing (RNA-seq) was employed to investigate the transcriptional mechanisms. This analysis followed a 24-h exposure of LNCaP cells to 20 μM genistein or baicalein. Principal components analysis revealed clear separation of the treatment groups from controls (Fig. S1A). Differential expression analysis identified 73 and 44 differentially expressed genes (DEGs) for genistein and baicalein, respectively (Table S2). Volcano plots visualized these distinct expression patterns (Fig. 1A). Heatmap analysis showed modulation of genes across functional modules including apoptosis, DNA repair, and PI3K–AKT signaling (Fig. 1B). KEGG enrichment analysis indicated a substantial association of baicalein-treated samples with p53 signaling, apoptosis, and nonhomologous end-joining (NHEJ) pathways (Fig. 1C). The corresponding KEGG pathway analysis for genistein-treated cells is provided in Fig S1B. Pathway-interaction network analysis further quantified the fold-change suppression of key functional modules by each flavonoid (Fig. 1D).

### Flavonoids sensitize LNCaP cells to ^177^Lu-PSMA617 and enhance related stress pathways

#### Synergistic cytotoxicity

**Fig. 2.**
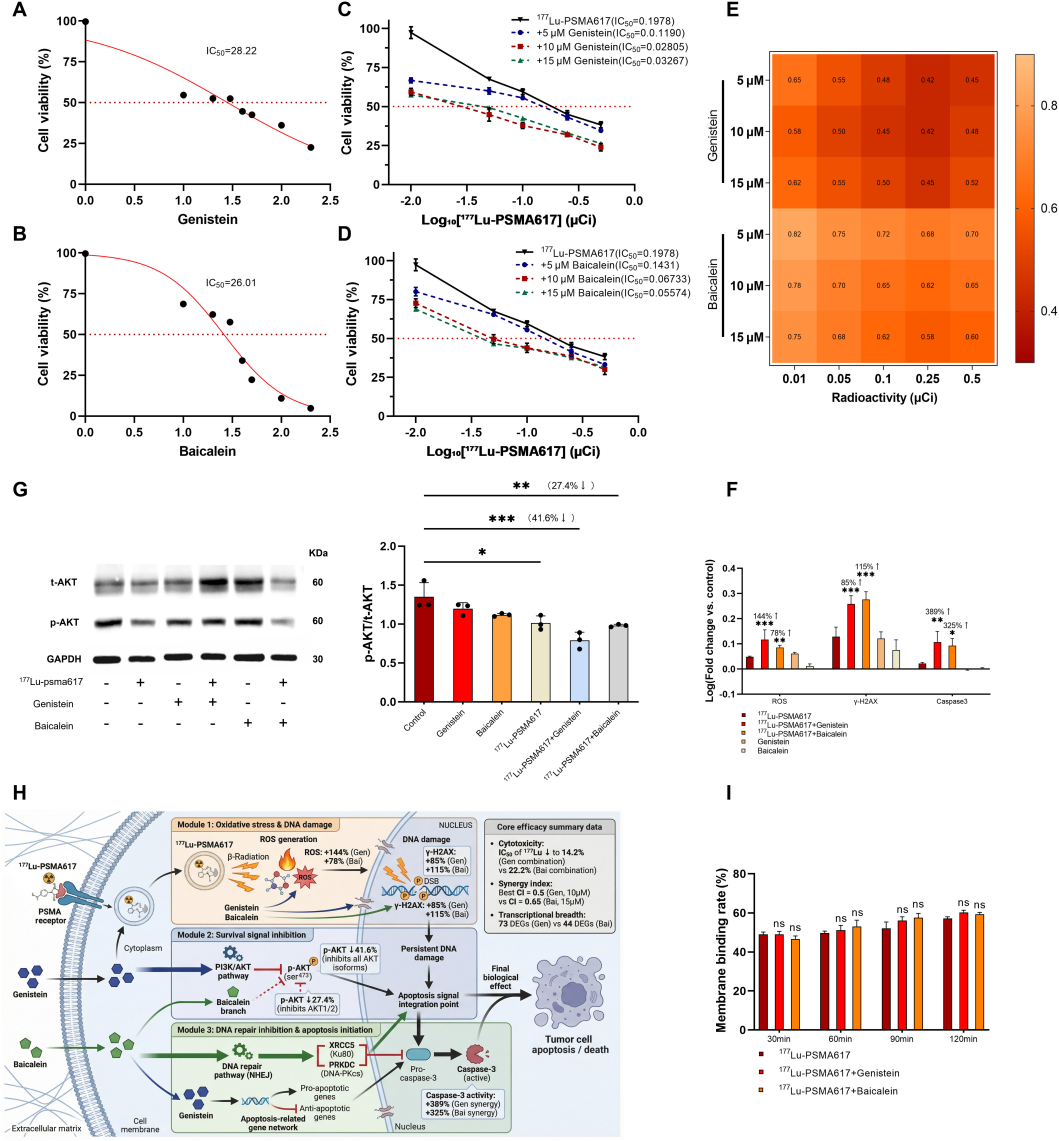
Enhanced radiosensitization and mechanistic insights of genistein and baicalein combined with ^177^Lu-PSMA617 in LNCaP cells. (A) Dose–response curve and IC_50_ value for genistein monotherapy. (B) Dose–response curve and IC_50_ value for baicalein monotherapy. (C) Dose–response curves of ^177^Lu-PSMA617 combined with increasing concentrations of genistein. Cotreatment reduced the IC_50_ of ^177^Lu-PSMA617 in a dose-dependent manner. (D) Dose–response curves of ^177^Lu-PSMA617 combined with increasing concentrations of baicalein. Cotreatment reduced the IC_50_ of ^177^Lu-PSMA617 in a dose-dependent manner. (E) Heatmap depicting combination index (CI) values derived using the Chou–Talalay method: CI < 0.8 indicates synergy. (F) Levels of ROS (left), γ-H2AX (middle), and cleaved caspase-3 (right). Combination treatments significantly elevated all markers versus corresponding monotherapies (*P* < 0.05). (G) Western blot analysis demonstrating that combination treatments significantly reduced the p-AKT/t-AKT ratio compared with control (*P* < 0.01). (H) Schematic model depicting the proposed multi-targeted synergistic mechanism of genistein and baicalein in enhancing ^177^Lu-PSMA617-induced cytotoxicity in LNCaP cells. (I) Membrane binding kinetics, which showed no significant effects or their interaction (*P* > 0.05). Data are presented as mean ± SD (*n* = 3 to 5 per group). ***P < 0.05, **P < 0.01, *P < 0.001 versus the indicated control or between groups as shown.

Genistein and baicalein monotherapy exhibited moderate cytotoxicity against human prostate cancer LNCaP cells, with median inhibitory concentration (IC_50_) values of 28.22 μM and 26.01 μM, respectively (Fig. 2A and B). The IC_50_ of ^177^Lu-PSMA617 alone was 7.32 kBq per well (Fig. 2C). Notably, cotreatment with low-toxicity concentrations (5 to 15 μM) of either flavonoid dose-dependently potentiated the cytotoxicity of ^177^Lu-PSMA617 (Fig. 2C and D). The most pronounced radiosensitization was achieved with 10 μM genistein, which reduced the IC_50_ of ^177^Lu-PSMA617 by 85.8% (to 1.04 kBq per well, 14.2% of the monotherapy value). Similarly, 15 μM baicalein reduced the IC_50_ by 77.8% (to 1.63 kBq per well, 22.2% of monotherapy). Formal synergy analysis confirmed these observations. All combination regimens yielded combination index (CI) values below 0.82, indicating synergistic interaction (Fig. 2E). The strongest synergy was observed for 10 μM genistein (CI = 0.5) and 15 μM baicalein (CI = 0.65).

#### Functional mechanisms underlying synergy

The synergistic cytotoxicity was associated with enhanced cellular stress and death pathways. Compared with ^177^Lu-PSMA617 monotherapy, cotreatment with 10 μM genistein or baicalein significantly increased intracellular reactive oxygen species (ROS) levels by 144% (*P* < 0.001) and 78% (*P* < 0.01), respectively (Fig. 2F, left). Levels of the DNA double-strand break marker γ-H2AX were elevated by 85% (genistein combination, *P* < 0.001) and 115% (baicalein combination, *P* < 0.001) (Fig. 2F, middle). Caspase-3 activity, a key apoptosis executor, was increased by 389% (genistein combination, *P* < 0.01) and 325% (baicalein combination, *P* < 0.05) (Fig. 2F, right). Western blot analysis further revealed that combination therapies inhibited a major cell survival pathway, significantly reducing the p-AKT(Ser^473^)/t-AKT ratio by 41.6% (genistein combination, *P* < 0.01) and 27.4% (baicalein combination, *P* < 0.01) compared with the control (Fig. 2G). A schematic model summarizing this multi-targeted synergistic mechanism of genistein and baicalein in enhancing ^177^Lu-PSMA617-induced cytotoxicity is presented (Fig. 2H).

### Flavonoids do not alter PSMA targeting of ^177^Lu-PSMA617

To rule out the possibility that the observed radiosensitization was due to altered targeting of ^177^Lu-PSMA617, we assessed its membrane binding kinetics in the presence of flavonoids. Two-way repeated-measures analysis of variance (ANOVA) revealed no statistically significant main effects of time or treatment group, nor any interaction between them (all *P* > 0.05; Fig. 2I). Post hoc tests confirmed that membrane binding did not differ significantly between treatment groups at any time point, indicating that the flavonoids did not interfere with the radiopharmaceutical’s binding to PSMA.

### Characterization and biodistribution of radiolabeled PSMA617

**Fig. 3.**
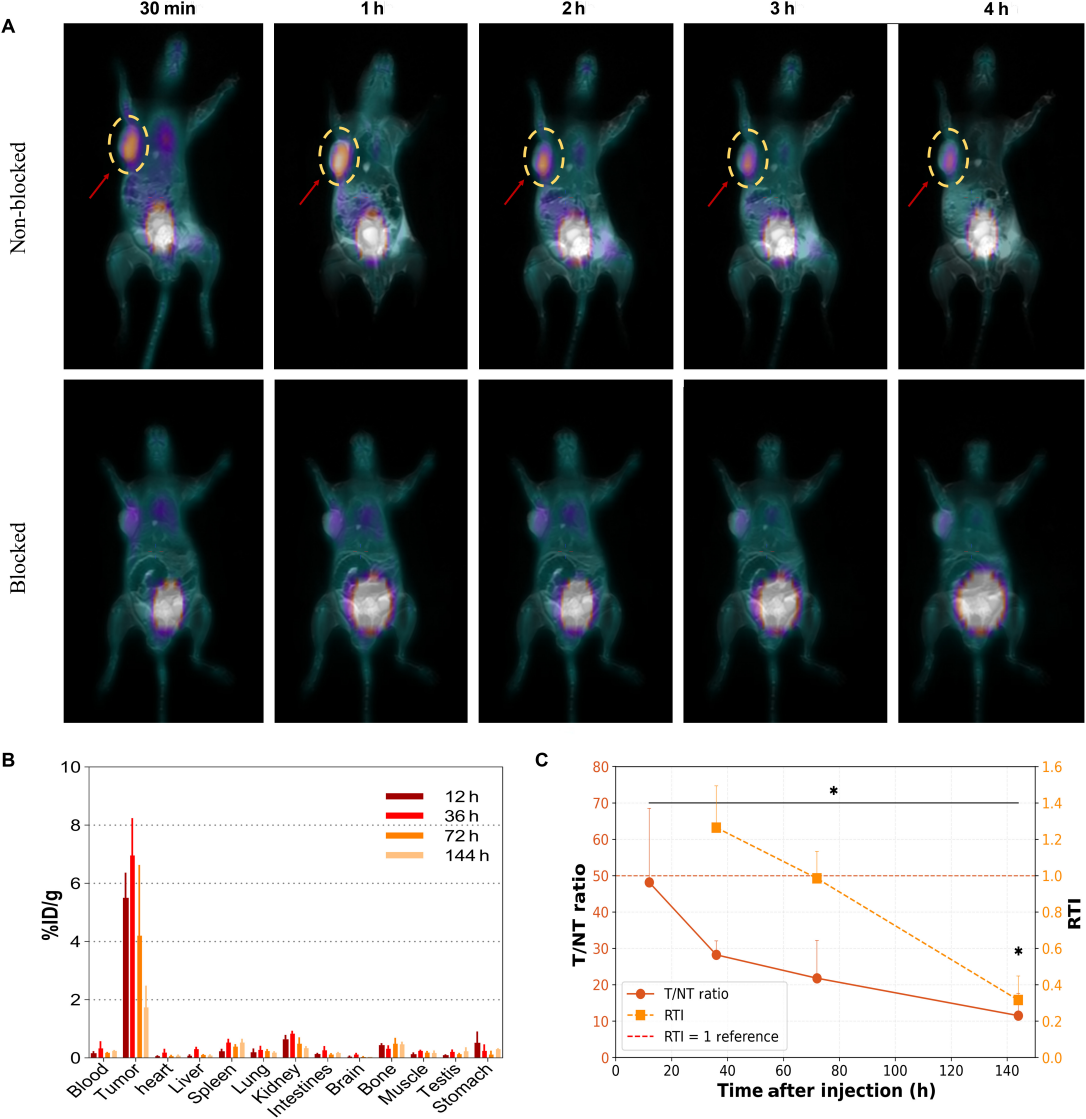
Biodistribution and pharmacokinetic profile of ^177^Lu-PSMA617. (A) Representative ^68^Ga-PSMA617 PET/MR images (4 h post-injection) in LNCaP tumor-bearing mice. Upper panel: Nonblocked group shows specific tumor uptake (arrow) with physiological renal/bladder excretion. Lower panel: Blockade with unlabeled PSMA617 markedly reduced tumor signal, confirming PSMA-specific targeting. (B) Tissue distribution of %ID/g in major organs at 12 to 144 h. Tumor uptake peaked at 36 h (6.96 ± 1.24 %ID/g) and decreased by 144 h (1.74 ± 0.75 %ID/g). Color scale: log_10_-transformed uptake. (C) Pharmacokinetic parameters. Left: Tumor-to-nontarget (T/NT) ratio was highest at 12 h (48.15 ± 20.35) and decreased significantly by 144 h (5.71 ± 0.56; *P < 0.05). Right: Retention index (RTI) remained high through 72 h (*P* > 0.05) before declining at 144 h (0.31 ± 0.13, *P* < 0.05). Data are mean ± SD (*n* = 3 per group). Significance is indicated as *P* < 0.05; ns, not significant.

Both ^177^Lu-PSMA617 and the imaging analog ^68^Ga-PSMA617 were successfully synthesized with high radiochemical purity (>99% after purification) and exhibited hydrophilic properties (LogD ≈ −1.15), as detailed in Fig S2. Positron emission tomography (PET)/magnetic resonance (MR) imaging with ^68^Ga-PSMA617 confirmed specific tumor uptake in LNCaP xenografts, which was blocked by excess unlabeled PSMA617 (Fig. 3A). Biodistribution studies of ^177^Lu-PSMA617 showed tumor uptake peaking at 36 h (6.96 ± 1.24 %ID/g) with favorable tumor-to-muscle ratios (Fig. 3B and C).

### In vivo antitumor efficacy and synergy

**Fig. 4.**
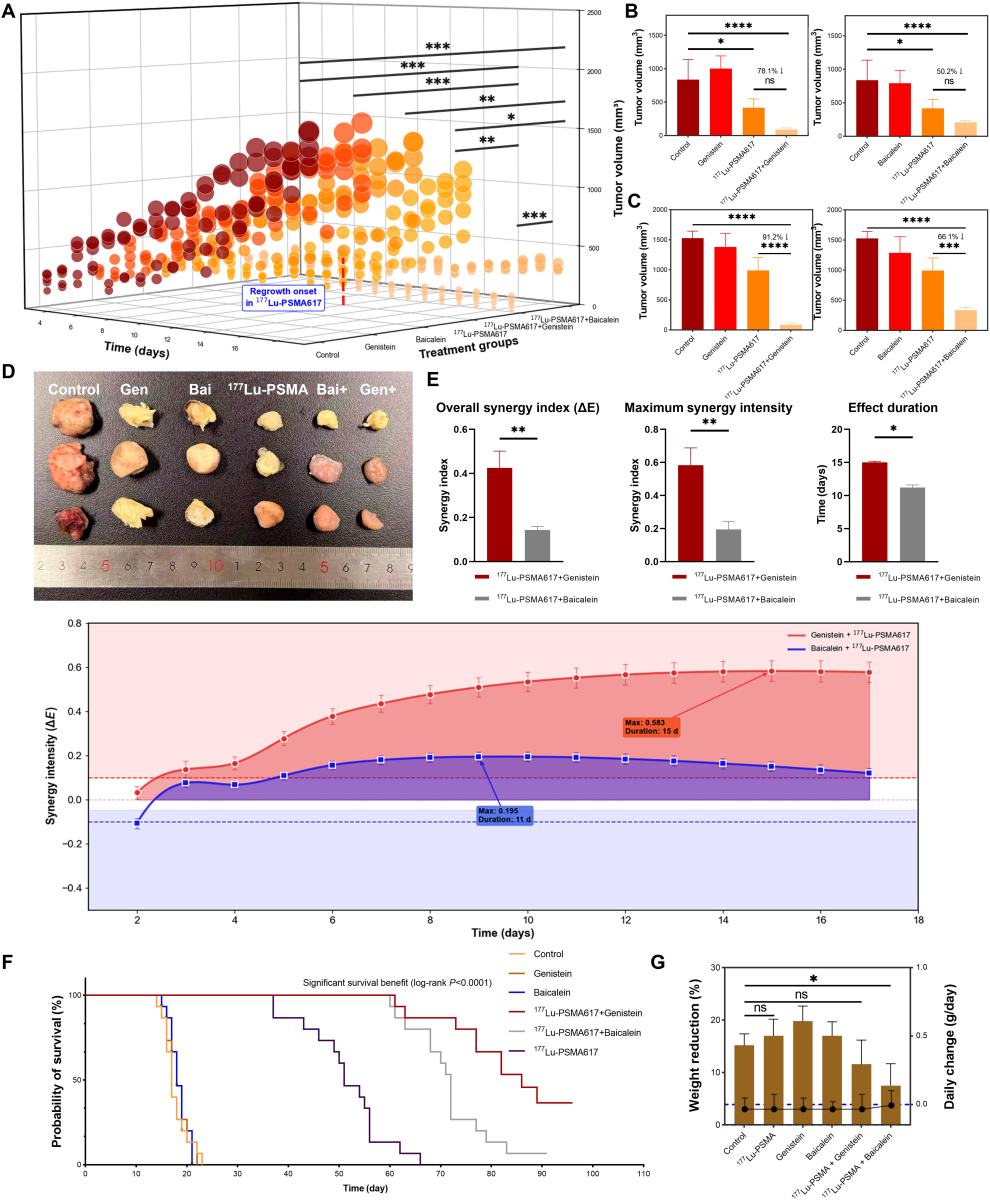
In vivo antitumor efficacy and synergy analysis. (A) Tumor growth curves over 17 d. The red dashed line indicates onset of regrowth in the monotherapy group. (B) Comparison of tumor volumes at day 11, prior to significant regrowth in the monotherapy group. (C) Terminal tumor volumes at day 17. Both combination therapies resulted in statistically significant tumor reduction compared to ^177^Lu-PSMA617 monotherapy. (D) Representative photographs of excised tumors. (E) Bliss synergy index (Δ*E*) over time. Δ*E* > 0.1 indicates synergy. (F) Kaplan–Meier survival curves. (G) Relative body weight change throughout the study. For (A) and (E), data points represent mean ± SEM (*n* = 5 mice per group per experiment). For (B) and (C), bars represent mean ± SD. Statistical significance is indicated as *P < 0.05, **P < 0.01, ***P < 0.001, ****P < 0.0001. Survival curves were compared using the log-rank test.

The in vivo efficacy of combination therapies was evaluated in LNCaP xenograft-bearing mice. Tumor growth curves (Fig. 4A) showed that both combination therapies trended toward better efficacy than monotherapies. To assess the early additive/synergistic effect prior to tumor regrowth in the monotherapy group, we compared tumor volumes at day 11. At this time point, the ^177^Lu-PSMA617 + genistein group exhibited a 78.1% reduction in tumor volume compared to ^177^Lu-PSMA617 monotherapy, while the ^177^Lu-PSMA617 + baicalein group showed a 50.2% reduction, demonstrating a strong trend of enhanced efficacy, although these differences did not reach statistical significance (*P* > 0.05) (Fig. 4B). By the study endpoint (day 17), the therapeutic benefit of the combinations became statistically profound. The ^177^Lu-PSMA617 + genistein group exhibited a 91.2% reduction in tumor volume compared to ^177^Lu-PSMA617 monotherapy (*P* < 0.0001), while the ^177^Lu-PSMA617 + baicalein group showed a 66.1% reduction (*P* < 0.001) (Fig. 4C). Representative photographs of excised tumors (Fig. 4D) illustrated the marked reduction in tumor size achieved by the combination therapies, consistent with the volumetric data. Quantitative assessment of drug interactions revealed distinct synergistic profiles between the 2 combination therapies (Fig. 4E). The genistein combination demonstrated superior synergistic properties, with an overall synergy index (Δ*E*) of 0.425, a maximal synergistic intensity of 0.583, and effective synergy maintained for 15 d. In comparison, the baicalein combination showed more modest effects, with an overall Δ*E* of 0.144, a peak intensity of 0.195, and an 11-d duration. Statistical analysis indicated no significant difference in the overall synergy indices between the 2 groups (*P* > 0.05).

### Survival benefit and safety profile

Kaplan–Meier survival analysis revealed a highly significant intergroup difference (log-rank test, *P* < 0.0001). Both combination therapies conferred a significant survival advantage over ^177^Lu-PSMA617 monotherapy (*P* < 0.0001), with the genistein combination showing a further benefit over the baicalein combination (*P* < 0.01) (Fig. 4F). In terms of safety, the combination therapies did not exacerbate the mild body weight loss associated with treatment (Fig. 4G).

**Fig. 5.**
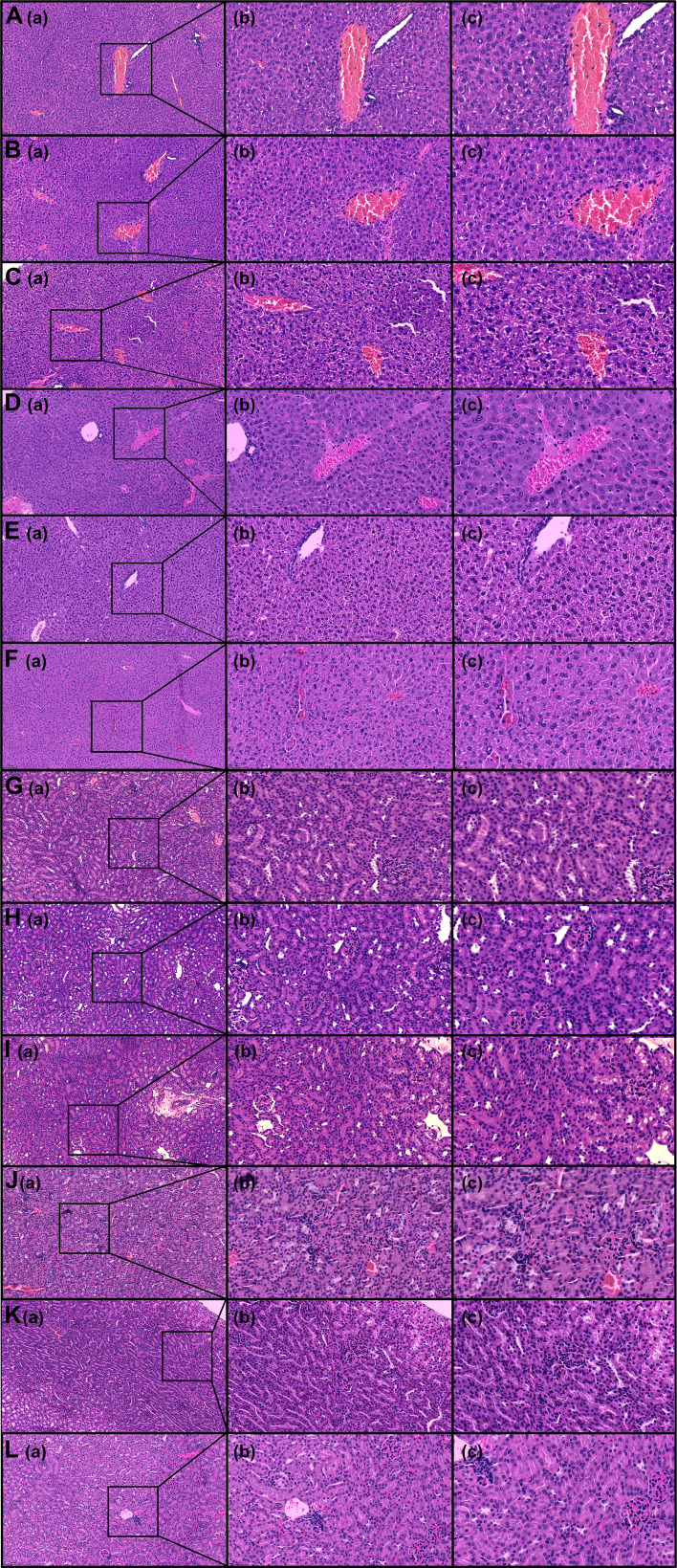
Assessment of treatment-related toxicity in liver and kidney tissues. (A to L) Representative H&E-stained sections of liver (A to F) and kidney (G to L) from treated mice. Liver sections (A to F): (A) Control group, showing normal hepatic architecture. (B) ^177^Lu-PSMA617 monotherapy group, with minimal changes (mild central venous congestion and scattered inflammation). (C) ^177^Lu-PSMA617 + genistein combination group. (D) ^177^Lu-PSMA617 + baicalein combination group. (E) Genistein monotherapy group. (F) Baicalein monotherapy group. Livers from combination and flavonoid-alone groups (C) to (F) remained largely intact, with occasional mild hepatocyte swelling noted in (C). Kidney sections (G to L): (G) Control group. (H) ^177^Lu-PSMA617 monotherapy group, showing mild nephrotoxicity (vacuolation and swelling of proximal tubular epithelial cells). (I) ^177^Lu-PSMA617 + genistein combination group. (J) ^177^Lu-PSMA617 + baicalein combination group. (K) Genistein monotherapy group. (L) Baicalein monotherapy group. Combination therapies (I and J) did not exacerbate renal damage compared to monotherapy (H). For each organ, images are presented at 3 magnifications: (a) low (20×), (b) medium (40×), and (c) high (60×). Scale bars, 100 μm (20×), 50 μm (40×), and 20 μm (60×).

Histopathological assessment of liver and kidneys indicated that ^177^Lu-PSMA617 monotherapy elicited mild renal tubular changes, but combination with either flavonoid did not aggravate injury in these organs (Fig. 5).

### Pathological analysis of tumor tissues

**Fig. 6.**
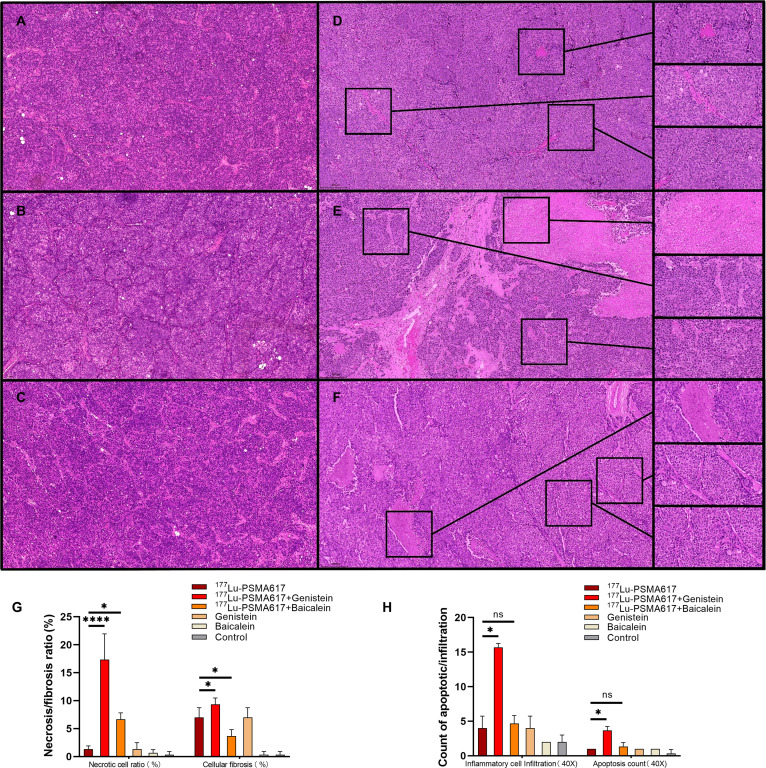
Histopathological analysis of tumor tissues following combination therapy. (A to F) Representative H&E-stained sections of LNCaP xenograft tumors. (A) Control (saline). (B) Genistein alone. (C) Baicalein alone. (D) ^177^Lu-PSMA617 alone, showing focal damage. (E) ^177^Lu-PSMA617 + genistein, showing extensive necrosis, fibrosis, and inflammation. (F) ^177^Lu-PSMA617 + baicalein, showing enhanced damage. (D to F) Left panel: overview (scale bar, 200 μm; 10× magnification); middle panel: intermediate-magnification view (scale bar, 100 μm; 20× magnification); right panels (top to bottom): high-magnification views of necrosis, fibrosis, and inflammation (scale bar, 50 μm; 40× magnification). (A to C) Single overview image is shown (scale bar, 200 μm). (G and H) Quantitative analysis of necrosis, fibrosis, inflammation, and apoptosis. Data are mean ± SD (*n* = 3). *P < 0.05, ****P < 0.0001.

Histopathological evaluation of tumor tissues revealed enhanced treatment effects in the combination groups. The ^177^Lu-PSMA617 + genistein group exhibited the highest proportion of necrosis, stromal fibrosis, inflammatory cell infiltration, and apoptotic cell count, all significantly exceeding other groups (*P* < 0.05) (Fig. 6). The ^177^Lu-PSMA617 + baicalein group also showed significantly enhanced necrosis compared to monotherapy.

## Discussion

This study demonstrates that the natural flavonoids genistein and baicalein act as potent and safe multi-targeted radiosensitizers, effectively overcoming intrinsic resistance to ^177^Lu-PSMA617 RLT in prostate cancer models. While PSMA-targeted RLT has transformed the treatment landscape for mCRPC, its efficacy is often limited by heterogeneous PSMA expression, robust DNA repair, and activated prosurvival pathways. Our work introduces a promising combinatorial strategy that leverages the multi-targeted pharmacology of natural compounds—an approach increasingly recognized for its potential to improve therapeutic indices in oncology [19,20]. It is crucial to acknowledge that resistance to RLT is multifactorial, driven by mechanisms such as tumor hypoxia, cancer stem cells, and cell cycle redistribution [8–10]. While this study focuses on modulating DDR and prosurvival signaling, we recognize that a robust clinical strategy will likely necessitate combining these approaches with agents targeting other resistance pathways.

The core synergy we identified operates through a coordinated, self-reinforcing cycle. ^177^Lu-PSMA617 delivers targeted β radiation, inducing primary DNA double-strand breaks (DSBs) and ROS. Both flavonoids dramatically amplify this initial damage. Functionally, we observed a potent pro-oxidant shift, consistent with the known ability of polyphenols to disrupt redox homeostasis in cancer cells [21,22]. This approach of targeting ROS dynamics for therapeutic benefit is gaining broad validation, as exemplified by novel enzyme-mimetic small molecules capable of sophisticated, context-dependent ROS regulation [23]. Crucially, our compounds concurrently impair the cell’s capacity to repair this damage. Our transcriptomic data provide key mechanistic insight: baicalein induced a targeted reduction in the expression of core NHEJ factors, notably XRCC5 (Ku80) and PRKDC (DNA-PKcs). This targeted suppression of a primary DSB repair machinery represents a potent radiosensitizing strategy, as NHEJ is a key mediator of radioresistance [24,25]. The resulting impairment in DNA repair is further evidenced by the marked increase in persistent γ-H2AX foci, a hallmark of unrepaired DSBs. Our transcriptomic profiling offers mechanistic clarity at the upstream level: Baicalein specifically down-regulated core NHEJ factors, most notably XRCC5 (Ku80) and PRKDC (DNA-PKcs). These findings suggest that the observed elevation in γ-H2AX stems from a dual mechanism: the direct induction of DNA damage by radiation compounded by a compromised repair capacity. A fundamental distinction exists between radiosensitization for conventional external beam radiotherapy (EBRT) and that for continuous low-dose-rate RLT. EBRT delivers high-dose-rate radiation over minutes, inducing overwhelming DNA damage that saturates repair capacity. In contrast, ^177^Lu-PSMA617 delivers protracted β-irradiation over days to weeks, a regimen under which NHEJ becomes the predominant pathway for repairing sublethal DNA double-strand breaks. Our transcriptomic data demonstrate that baicalein selectively down-regulates core NHEJ components (PRKDC, XRCC5), while genistein broadly suppresses PI3K/AKT signaling, which is known to modulate NHEJ efficiency. Thus, the NHEJ pathway represents a specific molecular vulnerability of PSMA-RLT-exposed tumor cells. By directly inhibiting this repair axis, both flavonoids potentiate the cytotoxic effect of protracted β-irradiation through a mechanism distinct from that of classical EBRT radiosensitizers, which typically rely on acute DNA damage overload or cell cycle redistribution.

Simultaneously, both flavonoids converge on inhibiting critical prosurvival signaling. The significant reduction in phosphorylated AKT demonstrates effective blockade of the PI3K/AKT/mTOR (mechanistic target of rapamycin) axis—a central hub that promotes cell survival, proliferation, and therapy resistance in prostate cancer [26,27]. This dual action of amplifying DNA damage while simultaneously inhibiting both its repair and prosurvival signaling creates an insurmountable stress scenario for cancer cells, culminating in robust activation of the intrinsic apoptotic pathway, as evidenced by the dramatic increase in caspase-3 activity. The distinct transcriptomic profiles—baicalein’s focused “surgical strike” on NHEJ versus genistein’s broader “network-wide” modulation—highlight complementary strategies to achieve this lethal synergy and underscore the pleiotropic nature of flavonoid action [28]. These distinct profiles may be attributable to subtle structural variations. The unique 6-hydroxyl group on the A-ring of baicalein could confer higher affinity or specificity for binding to key DNA repair proteins such as DNA-PK, whereas genistein’s isoflavone core with a 4′-hydroxyl on the B-ring might facilitate broader, albeit weaker, interactions with multiple kinases and receptors. Direct target engagement studies (e.g., kinase activity assays and surface plasmon resonance) are required to confirm these hypotheses.

The translational promise of this mechanistic synergy was rigorously validated in vivo. Both combination regimens, particularly the one involving genistein, profoundly suppressed tumor growth and significantly extended survival. The superior overall in vivo efficacy of genistein may be attributed to its more favorable pharmacokinetic profile, including oral bioavailability and broader tissue distribution, which facilitates sustained multi-target engagement [29]. Although the low oral bioavailability of baicalein suggests that plasma levels achieved with a 50 mg/kg dose fall well short of the 15 μM effective concentration observed in vitro, the combination regimen exhibited significant in vivo efficacy. This paradox may be attributed to multifaceted mechanisms: Notably, in vivo metabolic conversion to compounds like baicalin could preserve or synergize with the parent drug’s radiosensitizing activity. Furthermore, the repeated administration schedule likely leverages pharmacokinetic accumulation and persistent modulation of the tumor microenvironment to overcome low systemic exposure, thus sustaining long-term antitumor effects. Importantly, this enhanced antitumor efficacy was achieved without compromising safety. Comprehensive histopathological assessment revealed no exacerbation of the mild, agent-related renal toxicity associated with ^177^Lu-PSMA617 monotherapy and no evidence of significant hepatic injury. However, it must be emphasized that our safety evaluation was limited to body weight and histopathology of liver and kidney over a short observation period. We did not assess hematological parameters (e.g., complete blood counts) or biochemical markers of organ function [e.g., alanine aminotransferase (ALT)/aspartate aminotransferase (AST) and creatinine (Cr)/blood urea nitrogen (BUN)], and the use of immunodeficient NCG (NOD-Prkdcem26Cd52Il2rgem26Cd22/Gpt) mice precluded evaluation of immune-related toxicities. Thus, the combination can only be described as “preliminarily well-tolerated” rather than “safe” at this stage. This favorable safety profile is a critical advantage, strongly supported by the long history of dietary consumption and extensive preclinical data on the low systemic toxicity of these compounds [30,31].

While pro-oxidant and radiosensitizing activities were evident in vitro at 5 to 15 μM, the well-characterized biphasic response of flavonoids necessitates caution in clinical translation [32]. Heterogeneous biodistribution in vivo risks exposing normal tissues or tumor subregions to subtherapeutic concentrations that could paradoxically promote cell survival, potentially compromising overall efficacy. These concerns underscore the imperative for rigorous pharmacokinetics/pharmacodynamics (PK/PD) characterization and optimized dosing schedules to maintain intratumoral drug levels strictly within the synergistic therapeutic window. Moreover, despite the convenience of oral delivery, extrapolating effective murine doses (e.g., 150 mg/kg genistein, 50 mg/kg baicalein) to humans is fraught with challenges stemming from poor oral bioavailability (5% to 10% for genistein, negligible for baicalein) and substantial first-pass metabolism [33]. To achieve clinically relevant systemic exposure, future strategies must likely pivot toward advanced drug delivery systems—such as nanoparticles or cocrystals—to overcome bioavailability barriers or, alternatively, explore parenteral routes for early-stage clinical proof-of-concept trials. From a clinical development perspective, this flavonoid-based strategy presents a compelling alternative to current approaches for enhancing PSMA-RLT, such as combinations with PARPis, next-generation androgen receptor signaling inhibitors (ARSIs) [34–36], or strategies that represent the active frontier in overcoming AR-driven resistance and tumor heterogeneity [37]. By comparison, genistein and baicalein offer the potential for broader applicability, a reduced toxicity profile, lower cost, and the convenience of oral administration. Their multi-targeted nature may also reduce the likelihood of resistance mechanisms commonly encountered with single-target agents [38,39].

In conclusion, our integrated study provides a direct comparative assessment that delineates distinct yet complementary profiles for genistein and baicalein as radiosensitizers for ^177^Lu-PSMA617 therapy. Genistein demonstrated optimal synergistic potency at lower concentrations, elicited a more robust ROS and apoptotic response, and delivered superior in vivo tumor control and survival benefit. These attributes align with its broad, network-wide modulation of cellular homeostasis, as revealed by transcriptomics. Baicalein, while exhibiting a comparatively more modest overall synergy in our models, mounted a focused and potent assault on specific death and repair pathways, most notably inducing significant down-regulation of key NHEJ components. This distinct, mechanism-driven profile presents its own strategic value. Therefore, while genistein emerges as a strong lead candidate for immediate clinical translation based on its comprehensive efficacy-safety profile and established pharmacology, baicalein represents a valuable mechanistically targeted agent with potential utility in specific contexts, such as tumors with defined repair pathway dependencies or in rational polytherapy regimens. The natural origin, oral bioavailability, multi-targeted action, and favorable preliminary safety of both compounds collectively underscore their potential to improve mCRPC therapy by mitigating the additive toxicities often associated with conventional combinations.

Several limitations of this study should be acknowledged. First, the dependence on a single PSMA-high LNCaP xenograft model limits the generalizability of our findings, as it may not fully recapitulate the heterogeneous PSMA expression profiles and genetic complexity of human mCRPC. Second, our safety assessment was limited: The use of immunodeficient NCG mice precluded evaluation of hematological toxicity, bone marrow suppression, and immunomodulatory effects, and we did not examine functional biochemical toxicity markers beyond histopathology. Third, the PSMA binding assay only examined acute interference (within 2 h); longer-term effects on PSMA expression or internalization kinetics cannot be excluded and warrant further investigation. Future research should prioritize several complementary directions. First, model diversification is essential, with validation in a broader panel of prostate cancer models, including PSMA-low, AR-negative, and p53-mutant lines such as PC-3 and DU145, as well as patient-derived xenografts (PDXs) to establish generalizability. Second, model diversification is essential, with validation in PSMA-low/-negative patient-derived xenografts (PDXs) and androgen receptor variant-expressing models to establish generalizability. Third, the extension of this flavonoid-based strategy to α-therapy should be investigated by assessing synergy with ^225^Ac-PSMA617, leveraging the complementary toxicity profiles of these 2 radionuclides. Fourth, combination with emerging therapeutic strategies warrants exploration, particularly synergistic regimens that target the tumor microenvironment or utilize bio-responsive drug delivery systems [40,41]. Finally, early-phase clinical trials should be initiated, beginning with a phase I/IIa study of oral genistein as an adjuvant to standard-dose ^177^Lu-PSMA617, with hematological monitoring as a primary safety endpoint, followed by mechanism-driven evaluation of baicalein in appropriate molecular contexts.

## Materials and Methods

### Study design

The objective of this study was to systematically evaluate the synergistic effects and underlying mechanisms of 2 natural flavonoids, genistein and baicalein, in enhancing the efficacy of ^177^Lu-PSMA617 therapy for prostate cancer. The study employed a combination of in vitro and in vivo experiments. In vitro investigations utilized the human prostate cancer LNCaP cell line to assess cytotoxicity, membrane binding kinetics, ROS production, DNA damage (γ-H2AX foci), apoptosis (caspase-3 activity), and alterations in the AKT signaling pathway. Synergistic interactions were quantitatively analyzed using the Bliss independence model. All animal procedures were approved by the Animal Ethics Committee of the institution (protocol no. 2024AE01055). Six- to 7-week-old male NCG immunodeficient mice (GemPharmatech Co. Ltd.) were used to first confirm PSMA-specific targeting via ^68^Ga-PSMA617 PET/MR imaging and then to determine the biodistribution and pharmacokinetics of ^177^Lu-PSMA617 and to evaluate the antitumor efficacy, synergistic therapeutic effect, and systemic toxicity of the combination therapies compared to monotherapies and a control group. For the biodistribution study, mice (*n* = 3 per time point) were used. For the therapy efficacy study, we performed 3 independent experiments. In each experiment, 30 mice were randomly allocated into 6 groups (*n* = 5 per group per experiment). Data from all 3 experiments were pooled for survival analysis (final *n* = 15 per group), while tumor growth and body weight data were analyzed accounting for the repeated measures design within each experiment.

### Radiolabeling and purification of PSMA617

The labeling reaction was performed in a 5-ml vial. The following reagents were added sequentially: 100 μl of 0.1 M ammonium acetate buffer (pH 5.0), 12 μg of PSMA617 (Beijing Acima Pharmaceutical Technology Co. Ltd., Beijing, Tongzhou, China; catalog no. P751811), and 2 μl of ^177^LuCl₃ solution (37 MBq, 1 mCi, Chengdu New Radiomedicine Technology Medical Applications Co. Ltd., Nuoruite, Chengdu, China). The mixture reacted for 30 min in 70 °C. Labeling efficiency was analyzed using instant thin-layer chromatography (iTLC). Purification of the radiolabeled product was performed on a C18 cartridge (Waters Corporation, Milford, MA, USA; catalog no. WAT054960): 5-ml ethanol activation, 10-ml water rinsing, elution of the reaction product, and 10-ml water washing to remove free ^177^LuCl₃, and finally eluting with 500 μl of ethanol to obtain the purified product. The purified sample was dried to dryness at 70 °C and diluted to 500 μl with physiological saline for subsequent use. For ^68^Ga-PSMA617, the labeling was performed analogously, but with the reaction carried out in 0.1 M sodium acetate buffer (pH 4.0) at 90 °C for 10 min in the presence of ^68^GaCl_3_. Labeling efficiency was verified before and after purification using iTLC (Eckert & Ziegler Radiopharma GmbH Co. Ltd., Wilmington, MA, USA). Samples were spotted onto iTLC paper using sodium citrate buffer (pH 5.0) as the spot solution and developed with sodium citrate buffer (pH 5).

### Determination of the LogD value for ^177^Lu-PSMA617 and ^68^Ga-PSMA617

The lipophilicity (LogD) was determined using the shake-flask method. Briefly, ^177^Lu-PSMA617 or ^68^Ga-PSMA617 (20 μCi) was added to a mixture of 0.5 ml of phosphate-buffered saline (PBS) (pH 7.4) and 0.5 ml of saturated n-octanol. The mixture was vortexed thoroughly for 1 min and then centrifuged at 1,000 rpm for 10 min to achieve phase separation. Aliquots (100 μl) from the aqueous and organic phases were collected separately. Radioactivity in each aliquot was measured using a radio-TLC (thinlayer chromatography) scanner (Model FCY-6112, Hefei Zhongcheng Electromechanical Technology Development Co. Ltd.). The LogD value was calculated as:

$$\text{LogD}=\log_{10}\left(\frac{C_{\text{org}}}{C_{\text{aq}}}\right)$$ (1)

### Cell culture and treatment

The human prostate cancer cell line LNCaP (Procell Life Science & Technology, Wuhan, China; catalog no. CL-0143) was used. Cells were authenticated by short tandem repeat (STR) profiling and regularly tested to confirm the absence of mycoplasma contamination. Cells between passages 5 and 15 were used for all experiments. Cells were cultured in RPMI 1640 medium (Procell; catalog no. CM-0143) supplemented with 10% fetal bovine serum and 1% penicillin/streptomycin at 37 °C in a 5% CO_2_ humidified incubator.

### RNA-seq analysis

Total RNA was extracted from LNCaP cells treated with genistein, baicalein (10 or 20 μM), or vehicle control for 24 h using TRIzol reagent (*n* = 3 biological replicates per group). RNA integrity and concentration were verified (Table S1). Sequencing libraries were prepared, and paired-end sequencing was performed on an Illumina platform. Bioinformatic analysis was conducted using Python. Raw reads were quality-trimmed and aligned to the human reference genome (GRCh38). Differential expression analysis was performed using the edgeR algorithm (via the edgeR package for Python), with genes exhibiting an absolute log₂ fold change > 0.59 and a Benjamini–Hochberg adjusted *P* value of <0.05 defined as significantly differentially expressed (DEGs). Functional enrichment analysis of Gene Ontology (GO) terms and Kyoto Encyclopedia of Genes and Genomes (KEGG) pathways was performed on the DEGs.

### Drug treatment

For monotherapy studies, genistein (1, 10, 20, 30, 50, 100, and 200 μM; Macklin Inc., Shanghai, China; catalog no. G810424), baicalein (1, 10, 20, 30, 50, 100, and 200 μM; Macklin Inc., Shanghai, China; catalog no. B802462), and ^177^Lu-PSMA617 (0.37, 1.85, 3.7, 9.25, and 18.5 kBq per well) were dissolved in dimethyl sulfoxide (DMSO; Macklin, Shanghai, China; catalog no. D8418) to treat cells, with a final concentration of <0.1%. For combination studies, cells were treated with 5, 10, or 15 μM genistein or baicalein plus gradient concentrations of ^177^Lu-PSMA617 (0.37, 1.85, 3.7, 9.25, and 18.5 kBq per well; *n* = 5, biological replicates). The final concentration of DMSO in all treatment and control groups was maintained below 0.1%.

### Cell viability and in vitro cytotoxicity assay

Cell viability was assessed using a fluorescence-based assay (Resazurin; Cayman Chemical, Ann Arbor, MI, USA; catalog no. 14322). LNCaP cells (5 × 10^3^ cells per well) were seeded into black transparent-bottom 96-well plates (NEST Biotechnology Co. Ltd., Wuxi, Jiangsu, China; product no. 701401) and precultured for 24 h at 37 °C in a 5% CO_2_ incubator. Subsequently, gradient concentrations of genistein, baicalein, or 0.1% DMSO were added for intervention and the cells were incubated for 48 h at 37 °C. The combination group received either 10 μM genistein or baicalein plus gradient concentrations of ^177^Lu-PSMA617 (*n* = 5, biological replicates). After treatment, 10 μl of resazurin working solution (Alamar Blue, Thermo Fisher Scientific, USA) was added to each well (final concentration 0.1 mg/ml in complete medium). Incubation continued for 2 h in the dark. Fluorescence intensity was measured using a microplate reader (FLX800, BioTek Instruments Inc., Winooski, VT, USA) at an excitation wavelength of 540 nm and an emission wavelength of 590 nm.

Calculation formula:

$$\text{Survival rate (\%)}=\frac{\text{Exp. signal}-\text{Blank signal}}{\text{Ctrl. signal}-\text{Blank signal}}\times 100$$ (2)

Synergistic effect: CI was calculated using the Chou–Talalay method (CompuSyn software).

### Cell surface membrane binding assay

LNCaP cells (2 × 10^5^ per well) were plated in 24-well plates and maintained under standard conditions for 24 h. Thereafter, they were treated with 10 μM genistein or baicalein at 37 °C for 24 h and then exposed to ^177^Lu-PSMA617 (18.5 kBq per well) for 30, 60, 90, or 120 min. The control group received only ^177^Lu-PSMA617 (*n* = 3, biological replicates). The medium was removed, and cells were washed 3 times with ice-cold PBS. Add pH 2.5 glycine buffer to each well and incubate at 4 °C with gentle shaking for 2 min. Collect supernatant as membrane-bound fraction, wash with 200 μl of PBS, and combine liquids. Add 300 μl of RIPA buffer (Beyotime Biotechnology, Shanghai, China; catalog no. P0013B) to each well and incubate for 30 min. The mixture was centrifuged at 12,000*g* for 10 min, the supernatant was collected, and the radioactivity was counted using a radioimmunoassay scanner.

Calculation formula:

$$\text{Membrane binding rate (\%)}=\frac{\text{Binding count}}{\text{Binding count}+\text{Endocytosis count}}\times 100$$ (3)

### ROS detection

Seed LNCaP cells (2 × 10^4^ cells per well) into black transparent-bottom 96-well plates and culture under standard conditions for 24 h. Subsequently, 10 μM genistein, baicalein, or 9.25 kBq of ^177^Lu-PSMA617 was added. The combination group received 10 μM genistein or baicalein + 9.25 kBq of ^177^Lu-PSMA617 for 1 d. The control group received 0.1% DMSO (*n* = 5, biological replicates). After intervention, the drug-containing medium was removed, the wells were washed with PBS, and 100 μl of DCFH-DA ROS fluorescent probe working solution (final concentration 5 μM, prepared in phenol red-free medium) was added to each well. Incubation continued for 30 min under dark conditions. Fluorescence intensity was detected using a fluorescence microplate reader (FLX800, BioTek Instruments Inc., Winooski, VT, USA) at an excitation wavelength of 488 nm and an emission wavelength of 525 nm.

Calculation formula:

$$\text{Relative ROS content (\%)}=\frac{\text{Exp. signal}-\text{Blank signal}}{\text{Ctrl. signal}-\text{Blank signal}}\times 100$$ (4)

### DNA damage assay

Detection of H2AX (γ-H2AX) foci formation, a marker of double-strand breaks, was performed using immunofluorescence (DNA Damage Assay Kit by γ-H2AX Immunofluorescence, Beyotime Biotechnology, Shanghai, China; catalog no. C2035S). LNCaP cells were seeded at a density of 2 × 10^4^ cells per well in black-walled, transparent-bottom 96-well plates (Corning, NY, USA; catalog no. 3904) and precultured under standard conditions for 24 h. Subsequently, cells were treated for 24 h with 10 μM genistein, 10 μM baicalein, 9.25 kBq of ^177^Lu-PSMA617, or combinations thereof (genistein + ^177^Lu-PSMA617 or baicalein + ^177^Lu-PSMA617). The control group was treated with 0.1% DMSO (*n* = 3, biological replicates). After treatment, cells were fixed with fixative (catalog no. C2035S-1) for 20 min according to the kit instructions, washed with wash buffer (catalog no. C2035S-2), and blocked with immunofluorescence blocking buffer (catalog no. C2035S-3). Cells were then incubated with a rabbit monoclonal anti-γ-H2AX primary antibody (catalog no. C2035S-5) at 4 °C overnight. After washing, cells were incubated for 1 h at room temperature in the dark with the fluorescently labeled secondary antibody anti-rabbit γ-H2AX monoclonal antibody (catalog no. C2035S-4). Finally, after washing, fluorescence was read on a BioTek FLX800 instrument (BioTek Instruments Inc., Winooski, VT, USA) with excitation and emission wavelengths set at 494 and 516 nm, respectively.

Calculation formula:

$$\text{Relative DNA damage efficiency (\%)}=\frac{\text{Exp. signal}-\text{Blank signal}}{\text{Ctrl. signal}-\text{Blank signal}}\times 100$$ (5)

### Apoptosis analysis: Caspase-3 activity assay

LNCaP cells (2 × 10^5^ cells per well) were seeded into 24-well plates and cultured under standard conditions for 24 h. Cells were treated with 10 μM genistein and baicalein, 37 kBq of ^177^Lu-PSMA617 alone, or in combination for 24 h (*n* = 3 biological replicates). After lysis, the caspase-3 fluorescent substrate Ac-DEVD-AMC (MedChemExpress, Monmouth Junction, NJ, USA; catalog no. HY-P1003) was added to a final concentration of 1 mg/ml. Incubate in the dark for 1 to 2 h. Add 0.1% DMSO to the negative control group. Correct for blank solvent controls. Fluorescence intensity was detected using a fluorescence microplate reader (FLX800, BioTek Instruments Inc., Winooski, VT, USA) at an excitation wavelength of 354 nm and an emission wavelength of 442 nm.

Calculation formula:

$$\text{Relative caspase-3 activity (\%)}=\frac{\text{Exp. signal}-\text{Blank signal}}{\text{Ctrl. signal}-\text{Blank signal}}\times 100$$ (6)

### Western blot analysis

LNCaP cells (8 × 10^5^ cells per well) were seeded into 6-well plates and cultured under standard conditions for 48 h. Groups included single ^177^Lu-PSMA617, 10 μM genistein, 10 μM baicalein, 10 μM genistein + ^177^Lu-PSMA617, 10 μM baicalein + ^177^Lu-PSMA617, and untreated control. Incubated for 24 h (*n* = 3 biological replicates). Lyse cells on ice using RIPA lysis buffer containing protease and phosphatase inhibitors (50 mM tris–HCl pH 7.4, 150 mM NaCl, 1% NP-40, 0.5% sodium deoxycholate, 0.1% SDS). The lysate was sonicated (3 × 5s pulses, 20% amplitude) and centrifuged (14,000*g*, 15 min, 4 °C). Protein concentration was determined by means of the BCA assay and adjusted to 2 μg/μl. Sample preparation: Mix 30 μg of total protein with 1× protein loading buffer (Yeasen, catalog no. LT101S), boil for 5 min, and then cool on ice. Electrophoresis: Separate samples using a 10% sodium dodecyl sulfate–polyacrylamide gel electrophoresis (SDS-PAGE) gel with the Mini-PROTEAN Tetra electrophoresis system (Bio-Rad) at 80 V for the concentrating gel and 120 V for the separating gel. Transfer: Wet transfer to polyvinylidene difluoride (PVDF) membrane. p-AKT/t-AKT transfer conditions: 200 mA/90 min; GAPDH: 250 mA/45 min (transfer buffer: tris–glycine–methanol system). Transfer efficiency was verified by Ponceau S staining. Blocking: Block phospho-protein antibodies with 5% BSA (BioFroxx, catalog no. 4240GR250)/TBST; block total protein antibodies with 5% skim milk powder/TBST. Primary antibodies were diluted in blocking buffer as follows: phospho-AKT (Ser^473^) [Cell Signaling Technology (CST) #4060, 1:1,000], AKT (CST #4691, 1:1,000), and glyceraldehyde-3-phosphate dehydrogenase (GAPDH) (Proteintech #60004, 1:5,000), and incubated overnight at 4 °C. After washing, membranes were incubated with horseradish peroxidase (HRP)-conjugated anti-rabbit IgG secondary antibody (CST #7074, 1:5,000) for 1 h at room temperature. For GAPDH, incubation was performed for 1 h at room temperature. Secondary antibody: Incubate with HRP-labeled anti-rabbit IgG for 1 h at room temperature in the dark. Detection: Membranes were immersed in enhanced chemiluminescence (ECL) substrate (luminol:peroxidase = 1:1, Vazyme Biotech Co. Ltd., Nanjing, China; catalog no. E422-01). Chemiluminescent signals were detected with the Tanon 5200 imaging system.

### In vivo distribution and antitumor efficacy of ^177^Lu-PSMA617

All animal experiments were approved by the Animal Ethics Committee of Nanjing Drum Tower Hospital, The Affiliated Hospital of Nanjing University Medical School (protocol no. 2024AE01055). Five million LNCaP cells mixed with BD Matrigel (BD Biosciences) were subcutaneously injected into the flank of 6- to 7-week-old male NCG nude mice (body weight 18 to 20 g; *n* = 45; GemPharmatech Co. Ltd., Nanjing, China). These mice were allocated as follows: 3 mice for ^68^Ga-PSMA617 PET/MR imaging, 12 mice for the biodistribution study (*n* = 3 per time point at 12, 36, 72, and 144 h), and the remaining 30 mice for the initial therapy efficacy study. Tumor volume (*V*) was calculated using the formula for a prolate ellipsoid: *V* = 0.5 × length × width^2^, which is widely used for subcutaneous xenograft estimation.

### ^68^Ga-PSMA617 PET/MR imaging

To noninvasively assess the baseline targeting and biodistribution of the PSMA617 ligand, small-animal PET/MR imaging was performed using ^68^Ga-PSMA617. Nude mice bearing LNCaP xenografts were divided into 2 groups: a nonblocked (positive) group (*n* = 3) and a preblocked (negative) group (*n* = 3). Mice in the negative group received an intravenous co-injection of an excess of unlabeled PSMA617 ligand for competitive blockade. All animals received an intravenous injection of 3.7 to 5.5 MBq of ^68^Ga-PSMA617 via the tail vein. Static PET scans were acquired at 4 h post-injection under isoflurane anesthesia using a preclinical PET/MR system. Simultaneous MR imaging was performed for anatomical reference. Image analysis was performed by visual assessment of the co-registered PET/MR images to evaluate the qualitative distribution pattern and relative signal intensity in tumors and normal organs.

### In vivo distribution of ^177^Lu-PSMA617

Tumor-bearing nude mice (LNCaP xenografts, 200 to 400 mm^3^) were randomized into 4 groups (*n* = 3) administered a single intravenous injection of ^177^Lu-PSMA617 (3.7 MBq in saline) via the tail vein. Mice were euthanized, and samples were collected at 12, 36, 72, and 144 h post-administration. Tumor, heart, liver, spleen, lung, kidney, stomach, intestine, testis, bone, muscle, and brain tissue samples were collected, rinsed with PBS, and weighed. Blood samples were collected at each time point and weighed. Radioactivity in each tissue sample was measured using a radioimmunoassay scanner and corrected for radioactive decay relative to the administration time point.

Calculate standardized uptake rate:

$$\%\mathrm{ID}/g=\frac{\text{Tissue count}}{\text{Total count}}\times \frac{100}{\text{Tissue weight (g)}}$$ (7)

Use muscle as nontarget tissue to calculate tumor/nontarget ratio (T/NT):

$$T/\mathrm{NT}=\frac{\text{Tumor}\%\mathrm{ID}/g}{\text{Muscle}\%\mathrm{ID}/g}$$ (8)

$$\text{RTI}=\frac{\text{Tumor}\%\mathrm{ID}/g(t)}{\text{Tumor}\%\mathrm{ID}/g(12\;\text{h})}$$ (9)

### Radioactive combination therapy animal experiment

Mice were randomly divided into 6 groups (*n* = 5 per group per experiment). Treatment regimens were as follows: Control: saline orally (every 3 d) + saline intravenously (single dose on day 1); genistein: genistein 150 mg/kg, dissolved in saline, administered by oral gavage every 3 d; baicalein: baicalein 50 mg/kg, dissolved in saline, administered by oral gavage every 3 d; ^177^Lu-PSMA617: a single intravenous dose of ^177^Lu-PSMA617 (3.7 MBq in saline) on day 1; combination groups: respective flavonoid regimen (every 3 d) plus a single intravenous dose of ^177^Lu-PSMA617 on day 1. Flavonoid administration commenced 24 h prior to the radioligand injection (day 0) and continued every 3 d throughout the study period until the mice reached the humane endpoint. This regimen was designed to ensure both preloading for sensitization prior to radiation exposure and sustained modulation of survival/repair pathways post-irradiation. For the therapy efficacy study, a total of 30 mice were randomly allocated into 6 experimental groups (*n* = 5 per group) to ensure equal statistical power for comparing the primary endpoint of tumor growth inhibition among all treatments. Tumor volume and body weight changes were monitored daily in each group. Tumor volume was measured using a Vernier caliper and calculated according to the formula above. Body weight was recorded using an electronic balance. Data recording continued until mice reached the humane endpoint. A tumor volume exceeding 1,500 mm^3^ was set as one of the humane endpoints for euthanasia. For each sample, the tumor volume change rate relative to its baseline volume *V*₀ on day 1 was calculated at each time point:

$$\text{Change rate}\;\left(\%\right)=V_{1}/V_{0}\times 100$$ (10)

### Bliss independence model analysis

To quantify interactions in combination therapy, the Bliss independence model is employed. This model assumes that 2 drugs act independently. First, the theoretical expected effect *E* is calculated based on the single-drug effect scores *E*_A_ and *E*_B_, using the formula:

$$E_{\text{expected}}=E_{\text{A}}+E_{\text{B}}-\left(E_{\text{A}}\times E_{\text{B}}\right)$$ (11)

Subsequently, the experimentally measured combined effect is denoted as *E*_observed_, and the synergy index Δ*E* is defined as:

$$\Delta E=E_{\text{observed}}-E_{\text{expected}}$$ (12)

Based on the numerical range of Δ*E*, the following criteria are established:

ΔE>0.1:Synergy (13)

∣ΔE∣≤0.1:Additivity (14)

ΔE<−0.1:Antagonism (15)

### Experimental endpoints and pathological analysis

When mice reached humane endpoints, euthanasia was performed and tumor tissues were excised. Tumor samples underwent histopathological analysis. Tissue was fixed in 10% formalin, dehydrated with graded ethanol, paraffin-embedded, sectioned (5 μm), stained with hematoxylin and eosin (H&E), and visualized using a Nikon Eclipse E100 optical microscope. This assessed treatment effects on tumor morphology and cellular characteristics. Survival curves were plotted to analyze survival rate differences among groups.

### Statistical analysis

Data are presented as mean ± standard deviation (SD) from at least 3 independent biological replicates, unless otherwise indicated in the figure legends. All statistical analyses were performed using GraphPad Prism (GraphPad Software, San Diego, CA, USA) and Python (v3.x).

Normality of data distribution was evaluated with the Shapiro–Wilk test, and homogeneity of variances was assessed using the Brown–Forsythe test. For datasets violating the assumption of variance homogeneity—such as ROS levels, γ-H2AX foci counts, and caspase-3 activity shown in Fig. 2G—a natural logarithmic transformation was applied to meet parametric test requirements.

Statistical tests were selected based on data structure and variance characteristics. Longitudinal multi-group comparisons (e.g., tumor volume over time) were analyzed by 2-way repeated-measures ANOVA, followed by Tukey’s post hoc test when variances were equal or Games–Howell test when unequal. Single-time point multi-group data were evaluated using one-way ANOVA with Tukey’s test (homogeneous variances) or Dunnett’s T3 test (heterogeneous variances). For histopathological scoring (e.g., necrosis proportion), which typically exhibited heterogeneous variances, Welch’s ANOVA with Games–Howell post hoc testing was employed.

Drug interaction effects were quantified using 2 complementary approaches: the Chou–Talalay method (CompuSyn software) for in vitro CI calculation, and the Bliss independence model for in vivo synergy assessment (expressed as Δ*E*).

Survival analysis was conducted via Kaplan–Meier estimation. Group differences were tested using both the Mantel–Cox log-rank and Gehan–Breslow–Wilcoxon methods, with *P* values from pairwise contrasts adjusted for multiplicity using the Bonferroni correction.

To account for multiple correlated endpoints, the false discovery rate (FDR) was controlled at 5% using the 2-stage linear step-up procedure of Benjamini, Krieger, and Yekutieli.

## Data Availability

The data supporting the findings of this study are available within the article and its Supplementary Materials. The raw RNA-seq data generated in this study will be deposited in the Gene Expression Omnibus (GEO) upon article acceptance, with the accession number to be provided at that time. All other relevant data are available from the corresponding authors upon reasonable request.
