# Supplementary material for: Natural Flavonoids Genistein and Baicalein as Well-Tolerated Radiosensitizers to Enhance the Efficacy of 177Lu-PSMA617 in Prostate Cancer: In Vitro and In Vivo Studies
Source: Research (Wash D C). 2026 Jun 10;9:1314. doi: 10.34133/research.1314 (PMC13250281; doi:10.34133/research.1314)
Supplement: Supplementary 1 — Figs. S1 to S6 Tables S1 to S7 [file research.1314.f1.docx]

Title

Natural Flavonoids Genistein and Baicalein as Safe and Effective Radiosensitizers to Overcome Resistance to ^177^Lu-PSMA617 in Prostate Cancer: In Vitro and In Vivo Studies

**Authors**

Congjie He^1*^, Yuting Shao^1^, Ying Bao^2^, Jicong Li^2^, Songtao Xiao^2^, Hongwei Si^3^†, and Jian He^4^†

**Affiliations**

^1^ Department of Nuclear Medicine, Nanjing Drum Tower Hospital Clinical College of Nanjing University of Chinese Medicine, Nanjing, China.

^2^ Beijing Atomic Energy Institute, Beijing, China.

^3^ Department of Nuclear Medicine, The First Affiliated Hospital of Anhui Medical University, Hefei, China.

^4^ Department of Nuclear Medicine, Nanjing Drum Tower Hospital, Nanjing, China.

***Address correspondence to:** Hongwei Si, sihongwei@ahmu.edu.cn and Jian He, hjxueren@126.com.

SUPPLEMENTARY MATERIALS


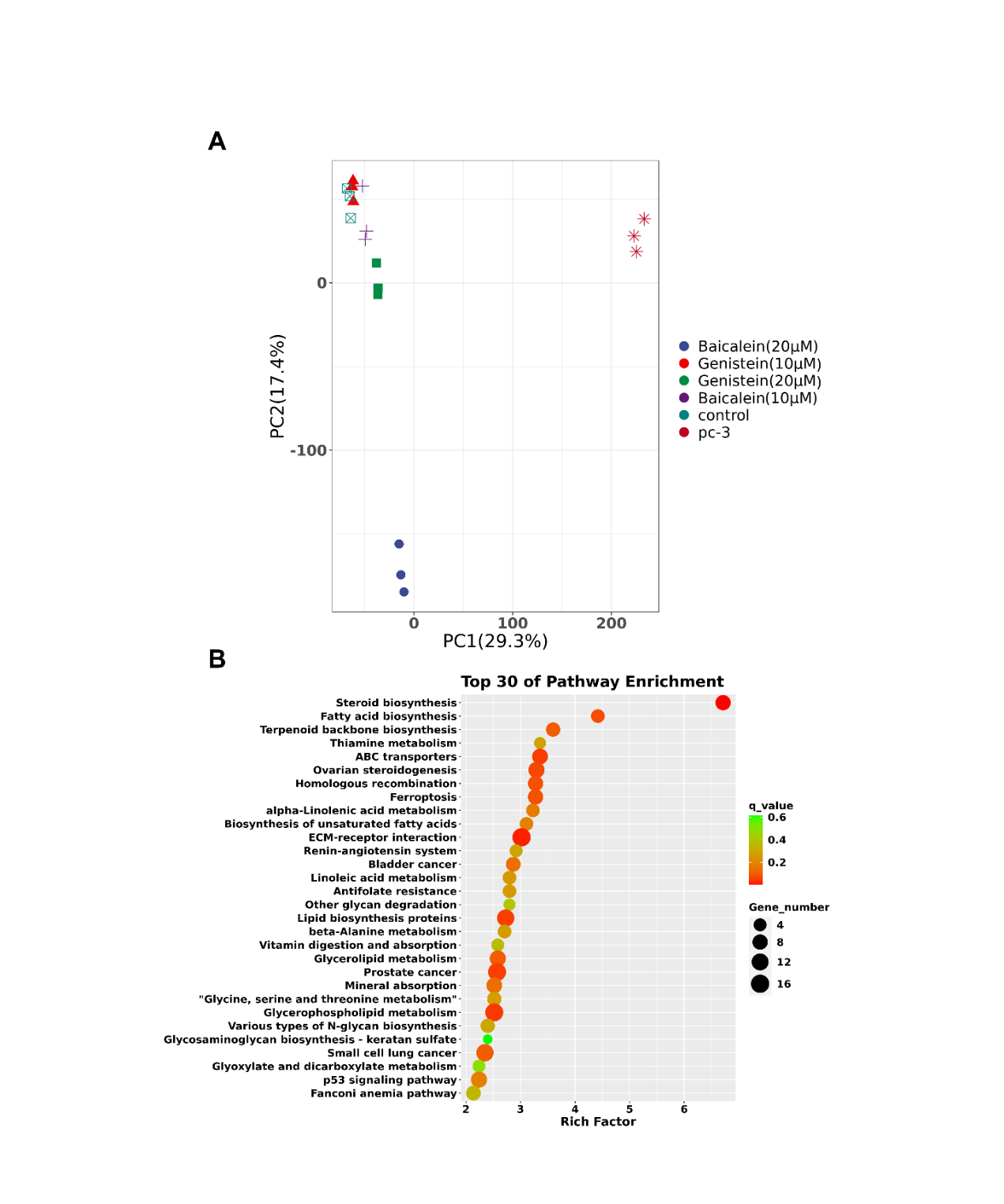


- ***Fig. S1.*** *Transcriptomic profiling supplementary data.****(A)****Principal component analysis (PCA) of RNA‑seq samples treated with 20 μM genistein or baicalein for 24 h.****(B)****KEGG pathway enrichment results for genistein‑treated cells (no terms reached q < 0.05). Differential expression was defined as |log₂FC| > 0.59 and adjusted p-value (q) < 0.05.*


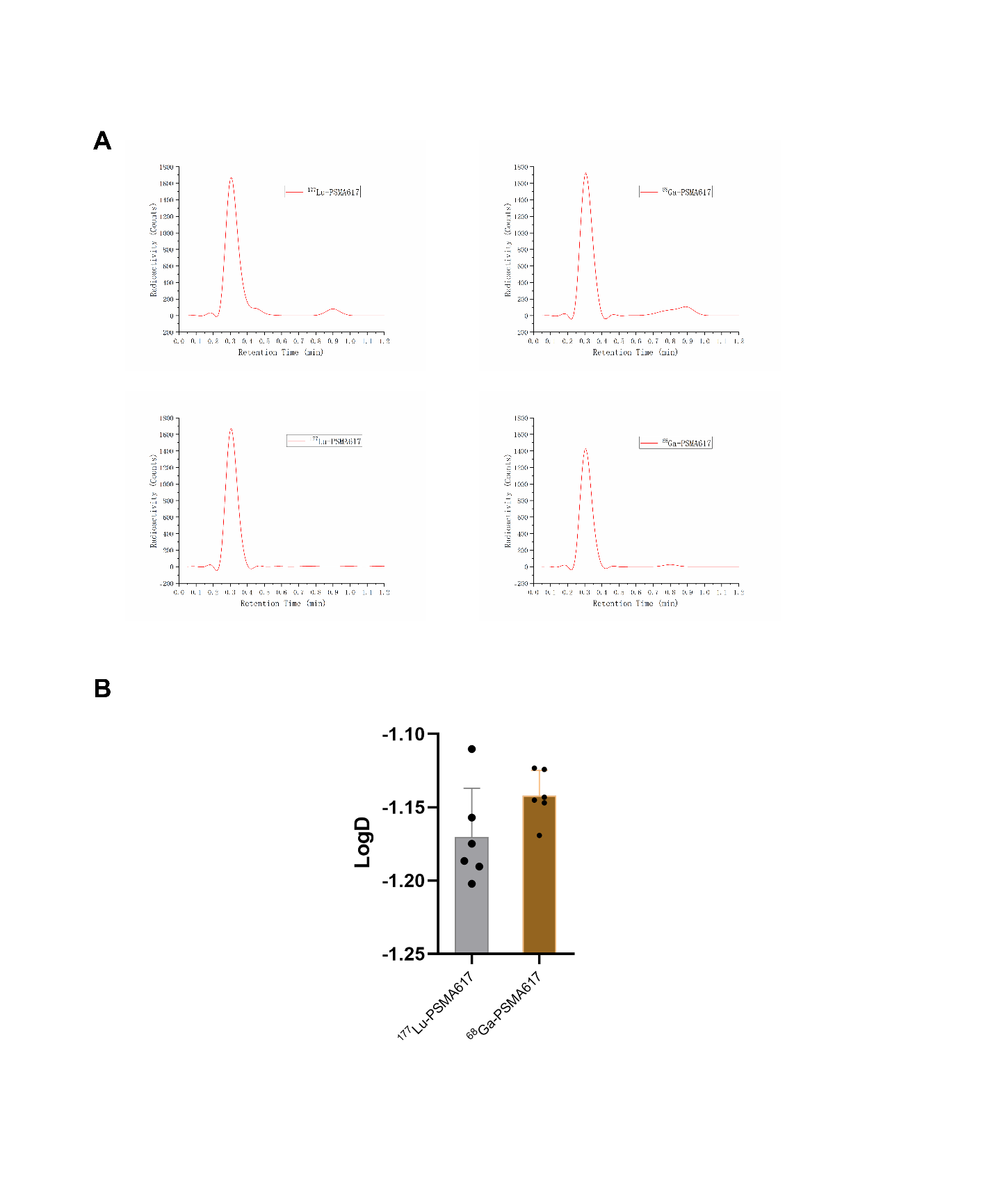


- ***Fig. S2.*** *Radiochemical characterization of ^177^Lu-PSMA617 and ^68^Ga-PSMA617.****(A)****iTLC analysis assessing radiochemical purity before and after purification. ^177^Lu-PSMA617(Left) and ^68^Ga-PSMA617(Right).* ***(B)****Determination of lipophilicity (LogD) via the shake-flask method. The LogD value for ^177^Lu-PSMA617 was -1.17 ± 0.033 (mean ± SD, n = 6), confirming its hydrophilic nature (LogD < 0, dashed line). The ^68^Ga-PSMA617 analogue exhibited a comparable LogD value of -1.14 ± 0.017 (n = 6).The dashed line at LogD = 0 represents the boundary between hydrophilicity (LogD < 0) and lipophilicity (LogD > 0).*


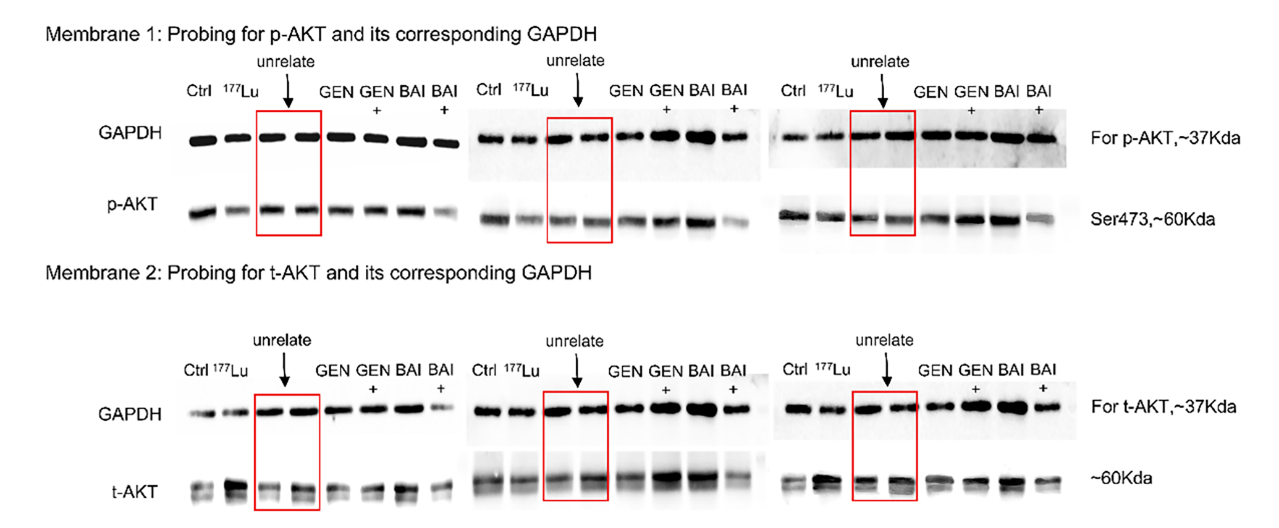


- ***Fig. S3.****Full-length western blot images corresponding to Figure 2G (p-AKT/t-AKT ratio) in the main text.(Membrane 1) Membranes probed for phosphorylated AKT (p-AKT, Ser473, ~60 kDa) and GAPDH (~37 kDa) as loading control.(Membrane 2) Membranes probed for total AKT (t-AKT, ~60 kDa) and GAPDH (~37 kDa).Lane assignments: 1 = Control (untreated); 2 = ^177^Lu-PSMA617 (0.5 μCi/well); 3–4 = Unrelated samples from different experiments; 5 = Genistein (10 μM); 6 = Baicalein (10 μM); 7 = ^177^Lu-PSMA617 + Genistein; 8 = ^177^Lu-PSMA617 + Baicalein.Red boxes indicate regions excluded from the main figure. Uncropped regions outside boxes represent primary data quantified in Figure 2G.*

***
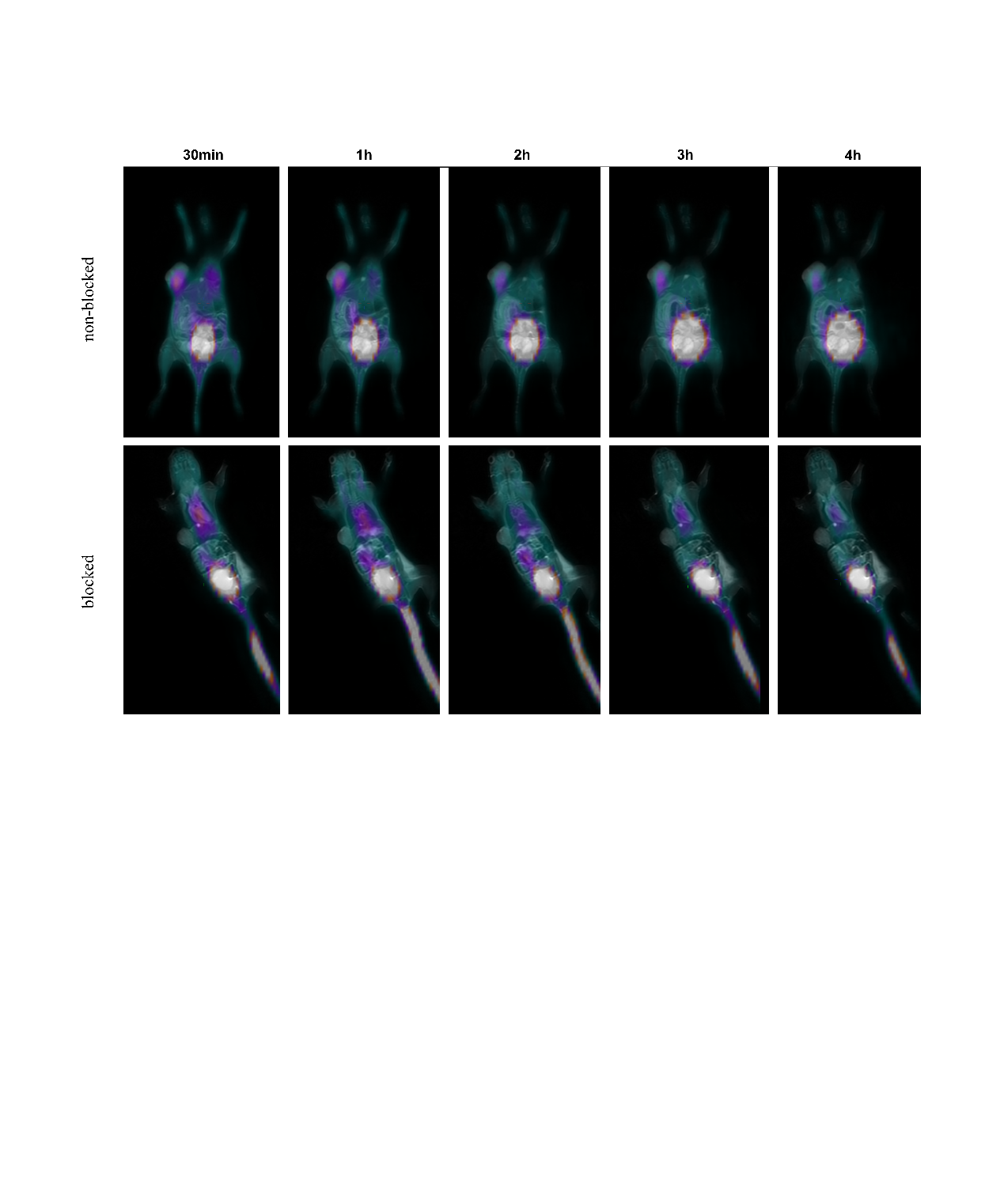
***

***
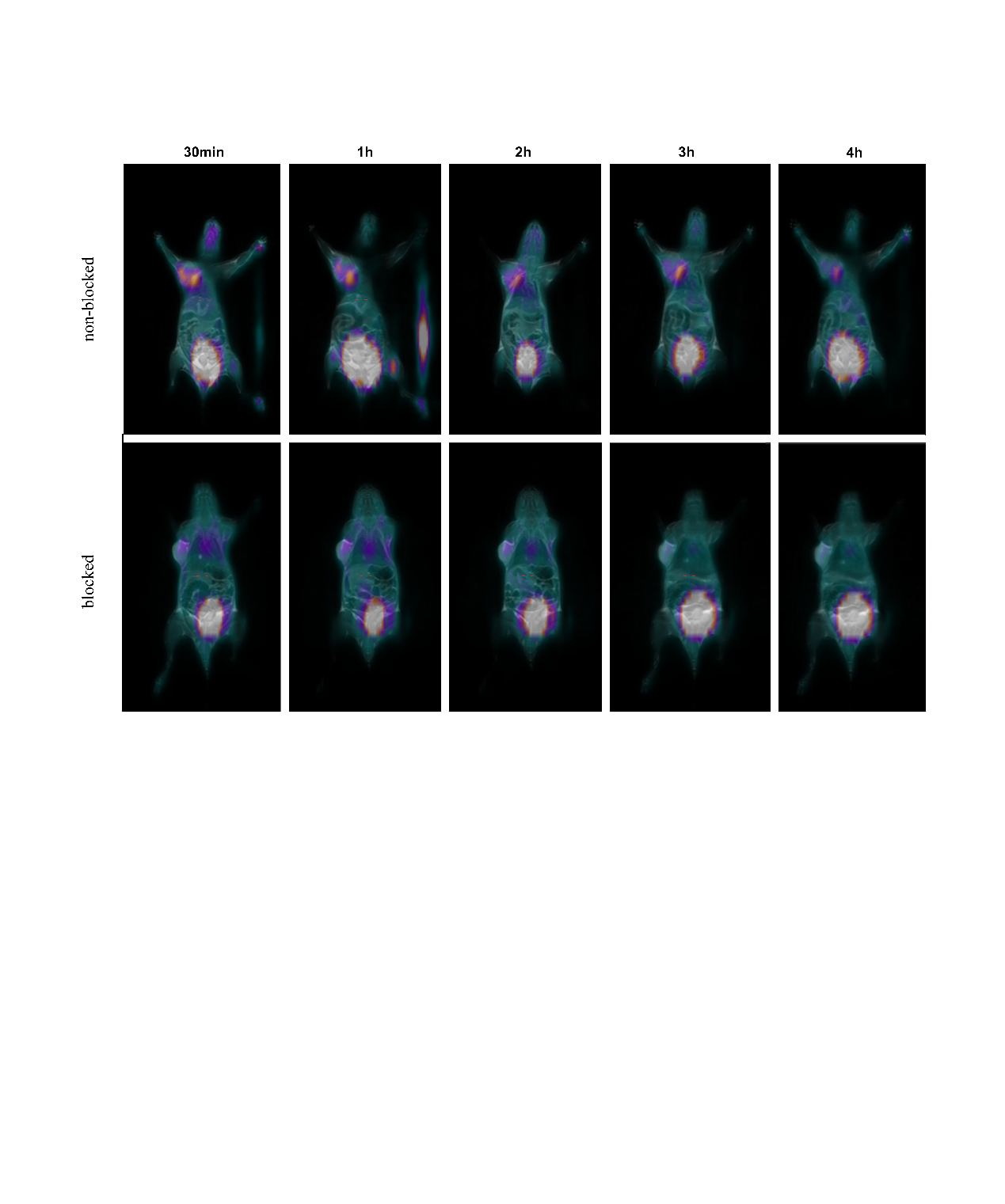
***

***Fig. S4.****In vivo PET/MR imaging confirming PSMA-specific targeting of ^68^Ga-PSMA617 (corresponding to Figure 3A in main text). Upper panel (Non-blocked group): Specific uptake of ^68^Ga-PSMA617 in the PSMA-expressing tumor (arrow) with physiological excretion via kidneys and bladder.Lower panel (Blocked group): Pre-injection of excess unlabeled PSMA-617 competitively abolished tumor signal, confirming target specificity.Images demonstrate the prerequisite PSMA-specific targeting for the subsequent ^177^Lu-PSMA617 therapy.*


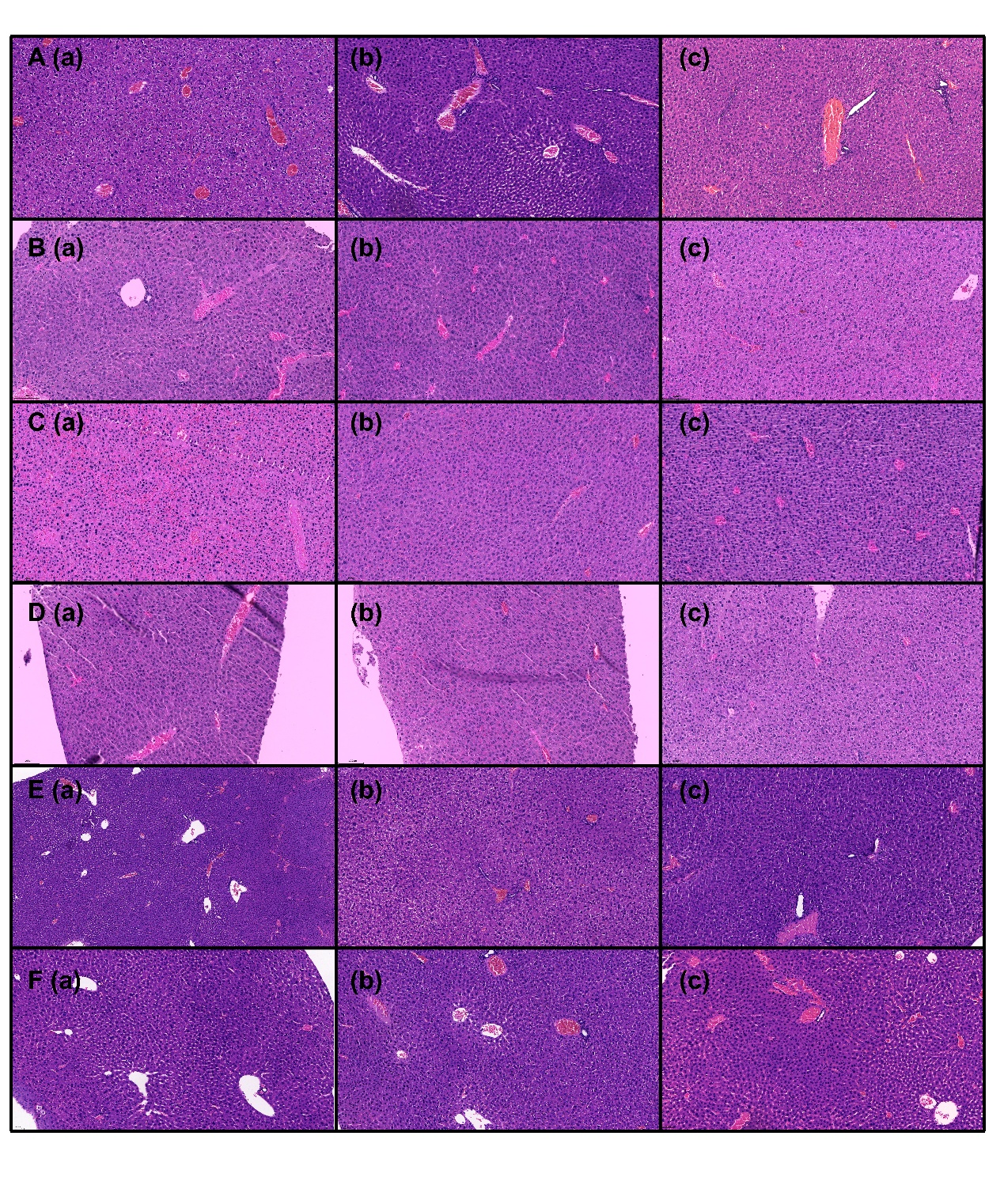


***
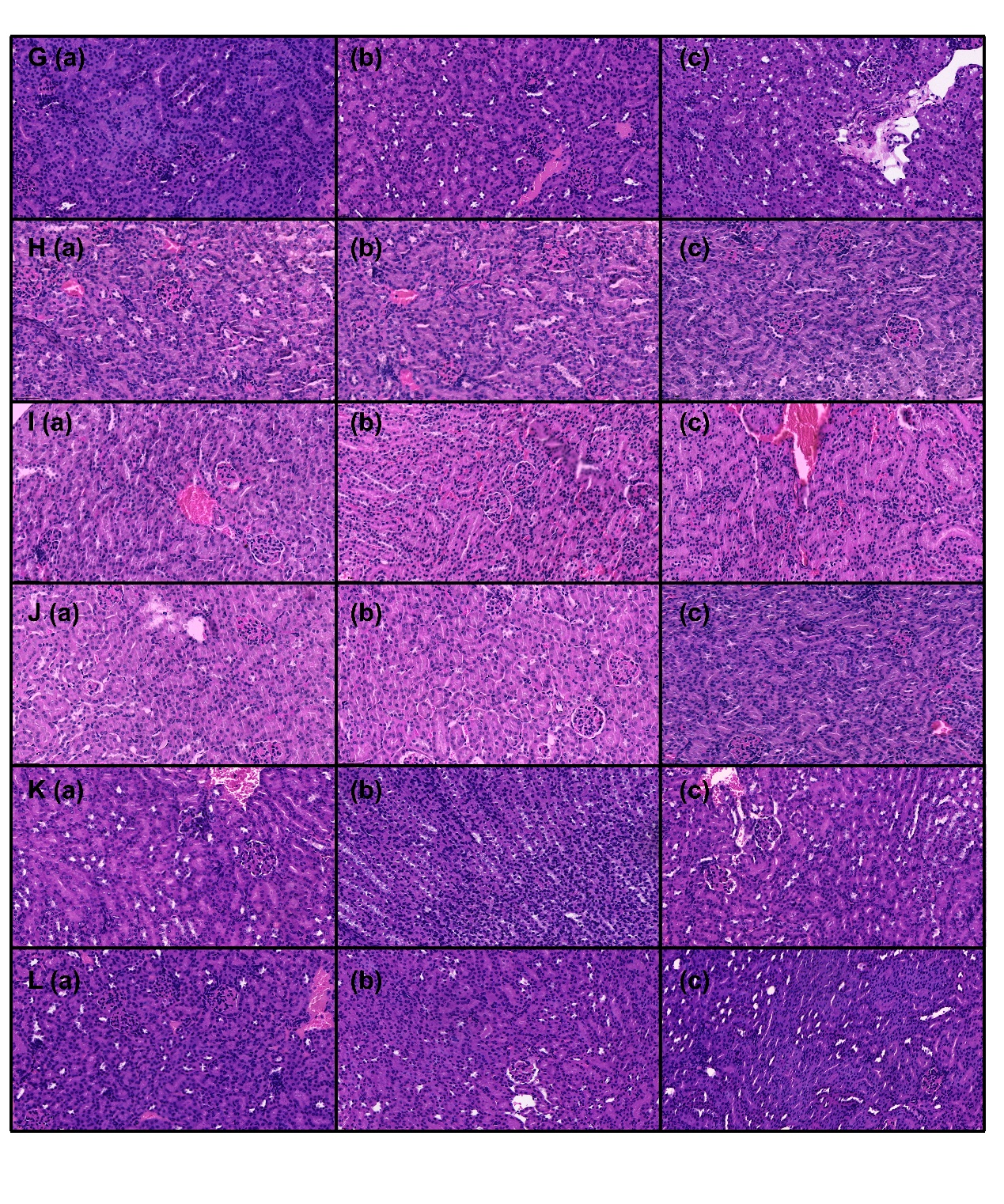
***

- ***Fig. S5.****Histopathological assessment of treatment-related toxicity in liver and kidney tissues (corresponding to Figure 5 in main text).Representative H&E-stained sections from mice in each treatment group.
  Liver (A–F): (A) Control, (B) ^177^Lu-PSMA617 monotherapy, (C) ^177^Lu-PSMA617 + Genistein, (D) ^177^Lu-PSMA617 + Baicalein, (E) Genistein alone, (F) Baicalein alone.
  Kidney (G–L): Same group order as liver.Scale bars: 50 μm (40x magnification for kidney), 100 μm (20x magnification for liver).*

**
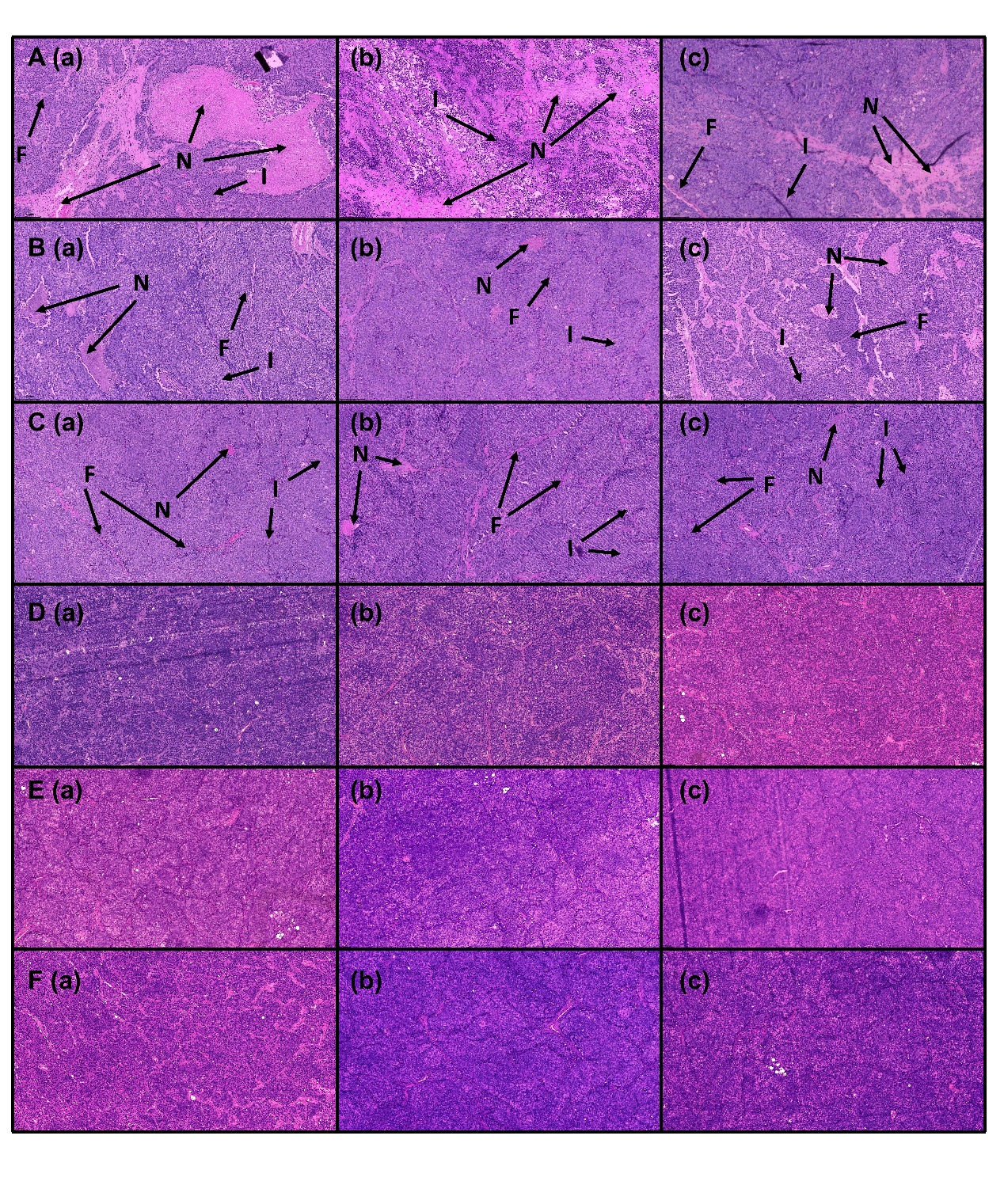
**

- ***Fig. S6.****Histopathological analysis of tumor tissues from all treatment groups****.*** *Representative hematoxylin and eosin (H&E)-stained sections (10× magnification, scale bar = 200 μm) from each mouse in every treatment group.* ***(A)*** *^177^Lu-PSMA617 + Genistein.* ***(B)*** *^177^Lu-PSMA617 + Baicalein.* ***(C)*** *^177^Lu-PSMA617 alone.* ***(D)*** *Control (saline).* ***(E)*** *Genistein alone.* ***(F)*** *Baicalein alone. Arrows indicate key histological features: N = necrosis, I = inflammatory cell infiltration, F = fibrosis.
  Groups: Control (saline-treated), Genistein (150 mg/kg every 3 days), Baicalein (50 mg/kg every 3 days), ^177^Lu-PSMA617 (100 μCi single dose), and combination therapies*
- ***Table S1.****Quality control metrics of total RNA samples used for RNA‑seq. Total RNA was extracted from LNCaP cells treated with Genistein or Baicalein at indicated concentrations. RNA quality was assessed by agarose gel electrophoresis and quantified using NanoDrop. A260/A280 ratio between 1.8-2.2 indicates high purity RNA.*

| Sample No. | Treatment Group | Concentration (ng/μL) | Volume (μL) | Total RNA (μg) | A260/A280 | Quality Result |
| --- | --- | --- | --- | --- | --- | --- |
| 001 | Untreated control | 204.2 | 15 | 3.06 | 2.06 | Qualified |
| 002 | Untreated control | 204.5 | 15 | 3.07 | 2.07 | Qualified |
| 003 | Untreated control | 196.8 | 15 | 2.95 | 2.08 | Qualified |
| 004 | 10 μM Genistein | 75.9 | 15 | 1.14 | 2.17 | Qualified |
| 005 | 10 μM Genistein | 78.0 | 15 | 1.17 | 2.10 | Qualified |
| 006 | 10 μM Genistein | 83.0 | 15 | 1.25 | 2.10 | Qualified |
| 007 | 10 μM Baicalein | 36.7 | 15 | 0.55 | 2.14 | Qualified |
| 008 | 10 μM Baicalein | 45.8 | 15 | 0.69 | 1.94 | Qualified |
| 009 | 10 μM Baicalein | 31.5 | 15 | 0.47 | 2.18 | Qualified |
| 010 | 20 μM Genistein | 87.8 | 15 | 1.32 | 2.06 | Qualified |
| 011 | 20 μM Genistein | 92.4 | 15 | 1.39 | 2.10 | Qualified |
| 012 | 20 μM Genistein | 86.2 | 15 | 1.29 | 2.05 | Qualified |
| 013 | 20 μM Baicalein | 43.4 | 15 | 0.65 | 2.10 | Qualified |
| 014 | 20 μM Baicalein | 63.9 | 15 | 0.96 | 2.10 | Qualified |
| 015 | 20 μM Baicalein | 43.1 | 15 | 0.65 | 2.07 | Qualified |

- ***Table S2.*** *Complete lists of differentially expressed genes (DEGs) for genistein and baicalein treatments (20 μM). Genes are ranked by P-value. log2FC: log2 fold change; Q-value: adjusted P-value; "inf" indicates infinite fold change due to zero expression in one condition. Genistein 20 μM top DEGs (73 genes); Baicalein 20 μM top DEGs (44 genes).*

**Genistein**

| Rank | Gene  ID | Gene  Symbol | Description | Locus | log2FC | P-value | Q-value | Direction |
| --- | --- | --- | --- | --- | --- | --- | --- | --- |
| 1 | ENSG00000170345 | FOS | Fos proto-oncogene, AP-1 transcription factor subunit [Source:HGNC Symbol;Acc:HGNC:3796] | 14:75278826-75282230:+ | 4.14487079087502 | 3.42236462591169e-44 | 9.897136261674e-40 | Up |
| 2 | ENSG00000120738 | EGR1 | early growth response 1 [Source:HGNC Symbol;Acc:HGNC:3238] | 5:138465479-138469303:+ | 3.15020335916076 | 2.26706247114921e-26 | 3.2780589801582e-22 | Up |
| 3 | ENSG00000125740 | FOSB | FosB proto-oncogene, AP-1 transcription factor subunit [Source:HGNC Symbol;Acc:HGNC:3797] | 19:45467995-45475179:+ | 4.21136403994202 | 1.65835534307968e-21 | 1.19894945416303e-17 | Up |
| 4 | ENSG00000268083 | - | novel protein | 19:38817471-38840178:- | 6.2292873286385 | 2.11785630448928e-20 | 1.22492572939051e-16 | Up |
| 5 | ENSG00000112245 | PTP4A1 | protein tyrosine phosphatase 4A1 [Source:HGNC Symbol;Acc:HGNC:9634] | 6:63521746-63583588:+ | 2.1878715269767 | 1.73512782726292e-13 | 6.27227020457705e-10 | Up |
| 6 | ENSG00000198932 | GPRASP1 | G protein-coupled receptor associated sorting protein 1 [Source:HGNC Symbol;Acc:HGNC:24834] | X:102651092-102659083:+ | 2.50020725839966 | 4.03477748813797e-11 | 1.16681730179462e-07 | Up |
| 7 | ENSG00000183506 | PI4KAP2 | phosphatidylinositol 4-kinase alpha pseudogene 2 [Source:HGNC Symbol;Acc:HGNC:33577] | 22:21473059-21488358:- | inf | 9.447828909552e-10 | 2.10170587873334e-06 | Up |
| 8 | ENSG00000180921 | FAM83H | family with sequence similarity 83 member H [Source:HGNC Symbol;Acc:HGNC:24797] | 8:143723933-143738234:- | 1.35828529097114 | 4.23090258250853e-09 | 8.15689811890427e-06 | Up |
| 9 | ENSG00000261796 | ISY1-RAB43 | ISY1-RAB43 readthrough [Source:HGNC Symbol;Acc:HGNC:42969] | 3:129087575-129161036:- | inf | 1.23279147979502e-08 | 1.98061648912179e-05 | Up |
| 10 | ENSG00000070404 | FSTL3 | follistatin like 3 [Source:HGNC Symbol;Acc:HGNC:3973] | 19:676392-683392:+ | 1.59982119380615 | 1.84397671376245e-08 | 2.66629812926482e-05 | Up |
| 11 | ENSG00000112237 | CCNC | cyclin C [Source:HGNC Symbol;Acc:HGNC:1581] | 6:99542387-99568825:- | 1.75124175452538 | 6.05216652033185e-08 | 8.33440969530842e-05 | Up |
| 12 | ENSG00000144118 | RALB | RAS like proto-oncogene B [Source:HGNC Symbol;Acc:HGNC:9840] | 2:120240064-120294710:+ | 1.68813900253899 | 9.99615432223969e-07 | 0.000996823402913274 | Up |
| 13 | ENSG00000124222 | STX16 | syntaxin 16 [Source:HGNC Symbol;Acc:HGNC:11431] | 20:58651272-58679526:+ | 1.85598280884648 | 4.96659848264318e-06 | 0.00377971214525153 | Up |
| 14 | ENSG00000198431 | TXNRD1 | thioredoxin reductase 1 [Source:HGNC Symbol;Acc:HGNC:12437] | 12:104215779-104350307:+ | 1.06678864436904 | 5.63849727388213e-06 | 0.00407649256658494 | Up |
| 15 | ENSG00000198824 | CHAMP1 | chromosome alignment maintaining phosphoprotein 1 [Source:HGNC Symbol;Acc:HGNC:20311] | 13:114314482-114337626:+ | 1.75647088239442 | 7.18466539935855e-06 | 0.0048319381089314 | Up |
| 16 | ENSG00000268713 | - | novel transcript | 19:57261354-57262738:+ | 2.43880542033384 | 1.34781501177683e-05 | 0.0082930770905477 | Up |
| 17 | ENSG00000117751 | PPP1R8 | protein phosphatase 1 regulatory subunit 8 [Source:HGNC Symbol;Acc:HGNC:9296] | 1:27830782-27851676:+ | 1.06487586727052 | 1.78642923201506e-05 | 0.010762863950134 | Up |
| 18 | ENSG00000108448 | TRIM16L | tripartite motif containing 16 like (pseudogene) [Source:HGNC Symbol;Acc:HGNC:32670] | 17:18722237-18735460:+ | 5.08660798822695 | 1.86754781707594e-05 | 0.0108015230644038 | Up |
| 19 | ENSG00000274049 | INO80B-WBP1 | INO80B-WBP1 readthrough (NMD candidate) [Source:HGNC Symbol;Acc:HGNC:49199] | 2:74455088-74460884:+ | inf | 2.11093328033455e-05 | 0.0116776643155069 | Up |
| 20 | ENSG00000279117 | - | TEC | 11:75260129-75262466:- | 2.87475537633603 | 2.14017154369745e-05 | 0.0116776643155069 | Up |
| 21 | ENSG00000125952 | MAX | MYC associated factor X [Source:HGNC Symbol;Acc:HGNC:6913] | 14:65006174-65102695:- | 1.04745333115497 | 3.76118816119698e-05 | 0.019423178648867 | Up |
| 22 | ENSG00000267216 | ZNF8-ERVK3-1 | ZNF8-ERVK3-1 readthrough (NMD candidate) [Source:HGNC Symbol;Acc:HGNC:56757] | 19:58278966-58315197:+ | 2.42470288812403 | 3.75288502225952e-05 | 0.019423178648867 | Up |
| 23 | ENSG00000197956 | S100A6 | S100 calcium binding protein A6 [Source:HGNC Symbol;Acc:HGNC:10496] | 1:153534599-153536244:- | 2.59781101695525 | 4.2571775198533e-05 | 0.0205938065052615 | Up |
| 24 | ENSG00000189403 | HMGB1 | high mobility group box 1 [Source:HGNC Symbol;Acc:HGNC:4983] | 13:30456704-30617597:- | 1.14925740969187 | 4.72372662233297e-05 | 0.0216834047922615 | Up |
| 25 | ENSG00000139372 | TDG | thymine DNA glycosylase [Source:HGNC Symbol;Acc:HGNC:11700] | 12:103965822-103988874:+ | 1.55503994543438 | 5.36227321723271e-05 | 0.0231187536146733 | Up |
| 26 | ENSG00000124243 | BCAS4 | breast carcinoma amplified sequence 4 [Source:HGNC Symbol;Acc:HGNC:14367] | 20:50794894-50877177:+ | 1.20080982410893 | 5.67629830935907e-05 | 0.0234504101154793 | Up |
| 27 | ENSG00000150764 | DIXDC1 | DIX domain containing 1 [Source:HGNC Symbol;Acc:HGNC:23695] | 11:111927144-112022653:+ | 1.56628503774447 | 6.19593110516285e-05 | 0.024886129393084 | Up |
| 28 | ENSG00000169891 | REPS2 | RALBP1 associated Eps domain containing 2 [Source:HGNC Symbol;Acc:HGNC:9963] | X:16946658-17153272:+ | 2.11276828542062 | 0.000102985172686735 | 0.0376990912522494 | Up |
| 29 | ENSG00000272772 | - | novel protein | 5:134157088-134225861:- | 6.71140387732109 | 0.000120276486529105 | 0.041906936312472 | Up |
| 30 | ENSG00000272256 | - | novel transcript, antisense to TMEM66 | 8:30082758-30083467:+ | 1.94937309161707 | 0.000119024186433801 | 0.041906936312472 | Up |
| 31 | ENSG00000063978 | RNF4 | ring finger protein 4 [Source:HGNC Symbol;Acc:HGNC:10067] | 4:2462220-2515857:+ | 1.43310044517006 | 0.000125204383524732 | 0.0421021577575782 | Up |
| 32 | ENSG00000293300 | - | novel transcript | 18:6462152-6463030:- | 5.27886801922484 | 0.000124354532353775 | 0.0421021577575782 | Up |
| 33 | ENSG00000289517 | - | novel protein | 22:42583836-42627688:- | -6.08215930276246 | 4.80792753230512e-19 | 2.31734093844553e-15 | Down |
| 34 | ENSG00000120903 | CHRNA2 | cholinergic receptor nicotinic alpha 2 subunit [Source:HGNC Symbol;Acc:HGNC:1956] | 8:27459756-27479883:- | -2.24994096081509 | 2.05811129355017e-12 | 6.61316894424192e-09 | Down |
| 35 | ENSG00000276043 | UHRF1 | ubiquitin like with PHD and ring finger domains 1 [Source:HGNC Symbol;Acc:HGNC:12556] | 19:4903080-4962154:+ | -1.64947770106431 | 3.89808700288251e-10 | 1.02480707305781e-06 | Down |
| 36 | ENSG00000163492 | CCDC141 | coiled-coil domain containing 141 [Source:HGNC Symbol;Acc:HGNC:26821] | 2:178829757-179050137:- | -5.41619361555455 | 8.81597308814253e-10 | 2.10170587873334e-06 | Down |
| 37 | ENSG00000196208 | GREB1 | growth regulating estrogen receptor binding 1 [Source:HGNC Symbol;Acc:HGNC:24885] | 2:11482341-11642788:+ | -1.45890194014685 | 2.0366947670562e-09 | 4.20708399774988e-06 | Down |
| 38 | ENSG00000100916 | BRMS1L | BRMS1 like transcriptional repressor [Source:HGNC Symbol;Acc:HGNC:20512] | 14:35826338-35932325:+ | -1.56237722891623 | 4.89811001955613e-09 | 8.85302772847148e-06 | Down |
| 39 | ENSG00000293552 | - | - | 9:6716157-7077263:+ | -4.62568665926246 | 1.12670568305984e-08 | 1.91665892049457e-05 | Down |
| 40 | ENSG00000269825 | - | novel zinc finger protein | 19:52650447-52690496:- | -4.86663452145838 | 1.40985430723656e-08 | 2.14587245847231e-05 | Down |
| 41 | ENSG00000139209 | SLC38A4 | solute carrier family 38 member 4 [Source:HGNC Symbol;Acc:HGNC:14679] | 12:46764761-46832408:- | -3.49383198717068 | 6.64094438556895e-08 | 8.72952139483039e-05 | Down |
| 42 | ENSG00000233802 | TRIM49D2 | tripartite motif containing 49D2 [Source:HGNC Symbol;Acc:HGNC:37217] | 11:89924064-89933063:+ | -1.23734407506139 | 1.07169167818691e-07 | 0.00013474892018038 | Down |
| 43 | ENSG00000198826 | ARHGAP11A | Rho GTPase activating protein 11A [Source:HGNC Symbol;Acc:HGNC:15783] | 15:32615144-32639941:+ | -1.74438851232529 | 1.74235179733523e-07 | 0.00020154828650855 | Down |
| 44 | ENSG00000145569 | OTULINL | OTU deubiquitinase with linear linkage specificity like [Source:HGNC Symbol;Acc:HGNC:25629] | 5:14581792-14616180:+ | -1.23654038961549 | 3.19162437399348e-07 | 0.000354994558736606 | Down |
| 45 | ENSG00000135686 | KLHL36 | kelch like family member 36 [Source:HGNC Symbol;Acc:HGNC:17844] | 16:84648511-84667686:+ | -1.4458592361066 | 9.64156070742293e-07 | 0.000996823402913274 | Down |
| 46 | ENSG00000128609 | NDUFA5 | NADH:ubiquinone oxidoreductase subunit A5 [Source:HGNC Symbol;Acc:HGNC:7688] | 7:123536997-123557904:- | -1.52699561734575 | 1.2688311887601e-06 | 0.00122311097159178 | Down |
| 47 | ENSG00000069206 | ADAM7 | ADAM metallopeptidase domain 7 [Source:HGNC Symbol;Acc:HGNC:214] | 8:24440930-24526970:+ | -1.33433658382889 | 1.47450108072292e-06 | 0.00137551925011052 | Down |
| 48 | ENSG00000175344 | CHRNA7 | cholinergic receptor nicotinic alpha 7 subunit [Source:HGNC Symbol;Acc:HGNC:1960] | 15:31923438-32173018:+ | -1.55582120382389 | 2.23233731083527e-06 | 0.00201740508412641 | Down |
| 49 | ENSG00000267064 | UXT-AS1 | UXT antisense RNA 1 [Source:HGNC Symbol;Acc:HGNC:49239] | X:47658833-47692326:+ | -2.05027922051077 | 2.52273102058513e-06 | 0.00221075328437277 | Down |
| 50 | ENSG00000290591 | - | - | 2:130012065-130034699:- | -2.58542257869527 | 2.88231163847833e-06 | 0.00245157559626925 | Down |
| 51 | ENSG00000198734 | F5 | coagulation factor V [Source:HGNC Symbol;Acc:HGNC:3542] | 1:169511951-169586588:- | -1.38452447805693 | 3.36800034978057e-06 | 0.00278283434615155 | Down |
| 52 | ENSG00000286379 | - | novel transcript | 1:36329630-36335406:+ | -2.34623542827808 | 3.52224899358267e-06 | 0.00282944218459492 | Down |
| 53 | ENSG00000286058 | - | novel transcript | 2:130026845-130051131:- | -1.50559463478266 | 3.84416407253647e-06 | 0.00300457785982925 | Down |
| 54 | ENSG00000018408 | WWTR1 | WW domain containing transcription regulator 1 [Source:HGNC Symbol;Acc:HGNC:24042] | 3:149517235-149736714:- | -1.29940683456564 | 5.49508087862701e-06 | 0.00407467292125678 | Down |
| 55 | ENSG00000223417 | TRIM49D1 | tripartite motif containing 49D1 [Source:HGNC Symbol;Acc:HGNC:43973] | 11:89911111-89922245:- | -1.08794692252733 | 6.37693119889206e-06 | 0.00449791398392096 | Down |
| 56 | ENSG00000144677 | CTDSPL | CTD small phosphatase like [Source:HGNC Symbol;Acc:HGNC:16890] | 3:37861880-37984469:+ | -1.13568252034551 | 7.04798208533033e-06 | 0.0048319381089314 | Down |
| 57 | ENSG00000101680 | LAMA1 | laminin subunit alpha 1 [Source:HGNC Symbol;Acc:HGNC:6481] | 18:6941742-7117797:- | -2.08222410459088 | 7.48629530464829e-06 | 0.00492036758898009 | Down |
| 58 | ENSG00000181904 | C5orf24 | chromosome 5 open reading frame 24 [Source:HGNC Symbol;Acc:HGNC:26746] | 5:134845680-134859735:+ | -1.45075134234453 | 8.1750058172835e-06 | 0.00525362207177825 | Down |
| 59 | ENSG00000183307 | TMEM121B | transmembrane protein 121B [Source:HGNC Symbol;Acc:HGNC:1844] | 22:17116297-17121367:- | -1.66208990122997 | 1.22157064689687e-05 | 0.0076796959864371 | Down |
| 60 | ENSG00000140262 | TCF12 | transcription factor 12 [Source:HGNC Symbol;Acc:HGNC:11623] | 15:56918623-57299281:+ | -1.16920392307592 | 1.83219589461452e-05 | 0.0108015230644038 | Down |
| 61 | ENSG00000167034 | NKX3-1 | NK3 homeobox 1 [Source:HGNC Symbol;Acc:HGNC:7838] | 8:23678697-23682938:- | -1.00345948507926 | 1.93644096906423e-05 | 0.0109803796832095 | Down |
| 62 | ENSG00000260719 | - | novel transcript, antisense to C16orf54 | 16:29745247-29748299:+ | -1.91873886361503 | 2.86698456705322e-05 | 0.0153537642027059 | Down |
| 63 | ENSG00000169641 | LUZP1 | leucine zipper protein 1 [Source:HGNC Symbol;Acc:HGNC:14985] | 1:23084030-23177808:- | -1.2910129785848 | 4.02524859721573e-05 | 0.0204221340671722 | Down |
| 64 | ENSG00000137877 | SPTBN5 | spectrin beta, non-erythrocytic 5 [Source:HGNC Symbol;Acc:HGNC:15680] | 15:41848146-41894053:- | -1.15836501638625 | 4.41514576342963e-05 | 0.0205938065052615 | Down |
| 65 | ENSG00000144218 | AFF3 | ALF transcription elongation factor 3 [Source:HGNC Symbol;Acc:HGNC:6473] | 2:99545419-100192428:- | -1.0714911140892 | 4.27350323961966e-05 | 0.0205938065052615 | Down |
| 66 | ENSG00000105429 | MEGF8 | multiple EGF like domains 8 [Source:HGNC Symbol;Acc:HGNC:3233] | 19:42325609-42378769:+ | -1.18547078464964 | 4.81138707499209e-05 | 0.0217407035658901 | Down |
| 67 | ENSG00000276418 | - | novel protein, TPD52-MRPS28 readthrough | 8:79918860-80080775:- | -2.15913997621034 | 5.43613280472279e-05 | 0.0231187536146733 | Down |
| 68 | ENSG00000075886 | TUBA3D | tubulin alpha 3d [Source:HGNC Symbol;Acc:HGNC:24071] | 2:131476119-131482934:+ | -2.28204221727051 | 5.62121214136906e-05 | 0.0234504101154793 | Down |
| 69 | ENSG00000188994 | ZNF292 | zinc finger protein 292 [Source:HGNC Symbol;Acc:HGNC:18410] | 6:87151803-87265943:+ | -1.37281986675201 | 5.98717394176306e-05 | 0.0243863497495558 | Down |
| 70 | ENSG00000235530 | TMEM11-DT | TMEM11 divergent transcript [Source:HGNC Symbol;Acc:HGNC:55671] | 17:21214322-21230035:+ | -2.19916580734933 | 8.43615383695032e-05 | 0.0321006753698377 | Down |
| 71 | ENSG00000178665 | ZNF713 | zinc finger protein 713 [Source:HGNC Symbol;Acc:HGNC:22043] | 7:55887456-55942530:+ | -1.2924587375021 | 0.000101119921455644 | 0.0374908590843049 | Down |
| 72 | ENSG00000274810 | NPHP3-ACAD11 | NPHP3-ACAD11 readthrough (NMD candidate) [Source:HGNC Symbol;Acc:HGNC:48351] | 3:132558142-132722459:- | -3.63719147996972 | 0.000118392056142233 | 0.041906936312472 | Down |
| 73 | ENSG00000276888 | - | novel transcript, antisense to POTEM | 14:19420975-19425017:+ | -1.03219222117921 | 0.000148297080282316 | 0.0492942903986699 | Down |

**Baicalein**

| Rank | Gene ID | Gene Symbol | Description | Locus | log2FC | P-value | Q-value | Direction |
| --- | --- | --- | --- | --- | --- | --- | --- | --- |
| 1 | ENSG00000255529 | POLR2M | RNA polymerase II subunit M [Source:HGNC Symbol;Acc:HGNC:14862] | 15:57706695-57782762:+ | 2.49840959371778 | 5.9270023410082e-15 | 5.62413252138268e-11 | Up |
| 2 | ENSG00000119396 | RAB14 | RAB14, member RAS oncogene family [Source:HGNC Symbol;Acc:HGNC:16524] | 9:121178133-121223014:- | 2.1280490457229 | 9.85817703218759e-13 | 7.0158181393821e-09 | Up |
| 3 | ENSG00000267645 | - | novel protein, POLR2J2-UPK3BL readthrough | 7:102637049-102671641:- | inf | 3.13016356012217e-12 | 1.78212732131996e-08 | Up |
| 4 | ENSG00000243414 | TICAM2 | TIR domain containing adaptor molecule 2 [Source:HGNC Symbol;Acc:HGNC:21354] | 5:115578496-115602479:- | 4.94126515212855 | 1.88276116101764e-10 | 8.93276032844821e-07 | Up |
| 5 | ENSG00000291315 | - | novel protein | 3:40312086-40312214:+ | 6.97263799707802 | 8.99276266812494e-09 | 2.55996974873513e-05 | Up |
| 6 | ENSG00000133818 | RRAS2 | RAS related 2 [Source:HGNC Symbol;Acc:HGNC:17271] | 11:14277922-14364506:- | 1.8006468379131 | 7.79273788485142e-09 | 2.55996974873513e-05 | Up |
| 7 | ENSG00000183506 | PI4KAP2 | phosphatidylinositol 4-kinase alpha pseudogene 2 [Source:HGNC Symbol;Acc:HGNC:33577] | 22:21473059-21488358:- | 4.23651681333859 | 2.54250162784045e-08 | 6.57976307633947e-05 | Up |
| 8 | ENSG00000076770 | MBNL3 | muscleblind like splicing regulator 3 [Source:HGNC Symbol;Acc:HGNC:20564] | X:132369320-132489968:- | 1.54656867634405 | 9.97666373161225e-08 | 0.000218465912652158 | Up |
| 9 | ENSG00000157224 | CLDN12 | claudin 12 [Source:HGNC Symbol;Acc:HGNC:2034] | 7:90383721-90513402:+ | 2.48778857399689 | 1.55450822939941e-07 | 0.000316087041187949 | Up |
| 10 | ENSG00000103066 | PLA2G15 | phospholipase A2 group XV [Source:HGNC Symbol;Acc:HGNC:17163] | 16:68245304-68261058:+ | 1.27989296704779 | 3.36683770220633e-07 | 0.00056378687569828 | Up |
| 11 | ENSG00000138459 | SLC35A5 | solute carrier family 35 member A5 [Source:HGNC Symbol;Acc:HGNC:20792] | 3:112561709-112585579:+ | 1.55845694821411 | 9.19766356786202e-07 | 0.00124680899422061 | Up |
| 12 | ENSG00000151835 | SACS | sacsin molecular chaperone [Source:HGNC Symbol;Acc:HGNC:10519] | 13:23288689-23433763:- | 1.90294144146264 | 2.68738232925983e-06 | 0.00318757136529332 | Up |
| 13 | ENSG00000285851 | - | novel transcript | 1:84498350-84556688:- | 1.77036120170188 | 4.65812641307672e-06 | 0.00510011094619442 | Up |
| 14 | ENSG00000168491 | CCDC110 | coiled-coil domain containing 110 [Source:HGNC Symbol;Acc:HGNC:28504] | 4:185445182-185471752:- | 1.97906529692752 | 8.6796477111285e-06 | 0.00915124190343315 | Up |
| 15 | ENSG00000181904 | C5orf24 | chromosome 5 open reading frame 24 [Source:HGNC Symbol;Acc:HGNC:26746] | 5:134845680-134859735:+ | 1.42398751039992 | 1.31707570543615e-05 | 0.0124977313688836 | Up |
| 16 | ENSG00000157600 | TMEM164 | transmembrane protein 164 [Source:HGNC Symbol;Acc:HGNC:26217] | X:110002631-110182734:+ | 1.7028568478642 | 1.56632208002509e-05 | 0.0143833840813143 | Up |
| 17 | ENSG00000205583 | STAG3L1 | STAG3 cohesin complex component like 1 (pseudogene) [Source:HGNC Symbol;Acc:HGNC:33852] | 7:75361374-75367087:+ | inf | 1.62382758078777e-05 | 0.0144454686694642 | Up |
| 18 | ENSG00000167766 | ZNF83 | zinc finger protein 83 [Source:HGNC Symbol;Acc:HGNC:13158] | 19:52594060-52690559:- | 1.06384398811137 | 1.69372783435649e-05 | 0.0145188479092832 | Up |
| 19 | ENSG00000138641 | HERC3 | HECT and RLD domain containing E3 ubiquitin protein ligase 3 [Source:HGNC Symbol;Acc:HGNC:4876] | 4:88592434-88708541:+ | 1.95716221200072 | 2.32188625118675e-05 | 0.0188848959750095 | Up |
| 20 | ENSG00000279117 | - | TEC | 11:75260129-75262466:- | 2.84572666065111 | 2.50300553919873e-05 | 0.0197925163012139 | Up |
| 21 | ENSG00000112237 | CCNC | cyclin C [Source:HGNC Symbol;Acc:HGNC:1581] | 6:99542387-99568825:- | 1.41682336307707 | 3.30892875913462e-05 | 0.0254581824287257 | Up |
| 22 | ENSG00000165156 | ZHX1 | zinc fingers and homeoboxes 1 [Source:HGNC Symbol;Acc:HGNC:12871] | 8:123248451-123275541:- | 1.29438919475008 | 3.75477401793265e-05 | 0.0271646630166688 | Up |
| 23 | ENSG00000138669 | PRKG2 | protein kinase cGMP-dependent 2 [Source:HGNC Symbol;Acc:HGNC:9416] | 4:81087370-81215222:- | 1.53767697101235 | 3.72108444927095e-05 | 0.0271646630166688 | Up |
| 24 | ENSG00000003989 | SLC7A2 | solute carrier family 7 member 2 [Source:HGNC Symbol;Acc:HGNC:11060] | 8:17497088-17570573:+ | 2.22995569851828 | 4.87940604072084e-05 | 0.0323028027351628 | Up |
| 25 | ENSG00000185596 | WASH3P | WASP family homolog 3, pseudogene [Source:HGNC Symbol;Acc:HGNC:24362] | 15:101966075-101976266:+ | inf | 5.14129131024878e-05 | 0.0332629863020118 | Up |
| 26 | ENSG00000146476 | ARMT1 | acidic residue methyltransferase 1 [Source:HGNC Symbol;Acc:HGNC:17872] | 6:151452258-151470101:+ | 1.32577635950462 | 5.58869623995639e-05 | 0.0353540924139641 | Up |
| 27 | ENSG00000106460 | TMEM106B | transmembrane protein 106B [Source:HGNC Symbol;Acc:HGNC:22407] | 7:12211270-12243367:+ | 1.0060979394862 | 7.2930548628701e-05 | 0.0423696719961884 | Up |
| 28 | ENSG00000260566 | - | novel transcript | 16:23061406-23064173:- | 1.24632873071619 | 7.60502223609864e-05 | 0.0427575382219949 | Up |
| 29 | ENSG00000136051 | WASHC4 | WASH complex subunit 4 [Source:HGNC Symbol;Acc:HGNC:29174] | 12:105107324-105169130:+ | 1.17527084081221 | 7.66021867187179e-05 | 0.0427575382219949 | Up |
| 30 | ENSG00000285943 | - | novel protein | 3:113361901-113515151:- | 1.56830173728524 | 8.62465383871397e-05 | 0.0472150040051289 | Up |
| 31 | ENSG00000269825 | - | novel zinc finger protein | 19:52650447-52690496:- | -5.08771201419052 | 4.95307716408033e-23 | 1.40999247629875e-18 | Down |
| 32 | ENSG00000070614 | NDST1 | N-deacetylase and N-sulfotransferase 1 [Source:HGNC Symbol;Acc:HGNC:7680] | 5:150485818-150558211:+ | -1.67300628065393 | 2.56703501620952e-10 | 1.04393979723481e-06 | Down |
| 33 | ENSG00000272589 | ZSWIM8-AS1 | ZSWIM8 antisense RNA 1 [Source:HGNC Symbol;Acc:HGNC:45103] | 10:73796514-73801399:- | -3.20193699910304 | 8.41055195600536e-09 | 2.55996974873513e-05 | Down |
| 34 | ENSG00000254806 | SYS1-DBNDD2 | SYS1-DBNDD2 readthrough (NMD candidate) [Source:HGNC Symbol;Acc:HGNC:33535] | 20:45363200-45410610:+ | -3.62331345780823 | 4.06173503308011e-08 | 9.63545093222429e-05 | Down |
| 35 | ENSG00000103353 | UBFD1 | ubiquitin family domain containing 1 [Source:HGNC Symbol;Acc:HGNC:30565] | 16:23557721-23574389:+ | -1.65507406909433 | 2.83203627270531e-07 | 0.000503872353594388 | Down |
| 36 | ENSG00000289517 | - | novel protein | 22:42583836-42627688:- | -6.04568132898713 | 2.81908022510446e-07 | 0.000503872353594388 | Down |
| 37 | ENSG00000072501 | SMC1A | structural maintenance of chromosomes 1A [Source:HGNC Symbol;Acc:HGNC:11111] | X:53374149-53422728:- | -1.23804318835977 | 4.35718585835309e-07 | 0.00068908894349854 | Down |
| 38 | ENSG00000228144 | - | novel protein | 12:66123917-66169985:- | -3.76189183483451 | 4.86203748926599e-07 | 0.000728461164247026 | Down |
| 39 | ENSG00000277194 | SNORD22 | small nucleolar RNA, C/D box 22 [Source:HGNC Symbol;Acc:HGNC:10145] | 11:62852910-62853035:- | -5.46239748258075 | 2.29350645467872e-06 | 0.00283866296718866 | Down |
| 40 | ENSG00000269955 | FMC1-LUC7L2 | FMC1-LUC7L2 readthrough [Source:HGNC Symbol;Acc:HGNC:44671] | 7:139341360-139422599:+ | -2.47012705766755 | 1.02767067492352e-05 | 0.0104481075368028 | Down |
| 41 | ENSG00000273294 | C1QTNF3-AMACR | C1QTNF3-AMACR readthrough (NMD candidate) [Source:HGNC Symbol;Acc:HGNC:49198] | 5:33987174-34124528:- | -4.24145335506994 | 1.7340809671396e-05 | 0.0145188479092832 | Down |
| 42 | ENSG00000259305 | ZHX1-C8orf76 | ZHX1-C8orf76 readthrough [Source:HGNC Symbol;Acc:HGNC:42975] | 8:123226189-123274284:- | -1.88523229768749 | 4.68301617251098e-05 | 0.0317408146149691 | Down |
| 43 | ENSG00000272410 | - | novel protein | 3:10249372-10285796:+ | -3.54827905453071 | 6.12947235324993e-05 | 0.0371250403148863 | Down |
| 44 | ENSG00000139531 | SUOX | sulfite oxidase [Source:HGNC Symbol;Acc:HGNC:11460] | 12:55997180-56006641:+ | -1.47262377639287 | 7.23610400538504e-05 | 0.0423696719961884 | Down |

- ***Table S3.*** *Cytotoxic effects of genistein and baicalein, alone and in combination with ^177^Lu-PSMA617 in LNCaP cells.Cell viability (%) was assessed after 48 h treatment using a resazurin-based assay. Data are mean ± SD of at least five independent experiments.*

| *Treatment* | *Concentration / Activity* | *Cell Viability (% Mean ± SD)* |
| --- | --- | --- |
| ***Genistein*** | *1 μM* | *101.4 ± 1.6* |
|  | *10 μM* | *54.7 ± 3.8* |
|  | *20 μM* | *52.6 ± 10.4* |
|  | *30 μM* | *52.7 ± 3.6* |
|  | *40 μM* | *44.7 ± 1.6* |
|  | *50 μM* | *42.3 ± 3.2* |
|  | *100 μM* | *35.1 ± 2.6* |
|  | *200 μM* | *22.5 ± 2.5* |
| ***Baicalein*** | *1 μM* | *101.6 ± 2.4* |
|  | *10 μM* | *68.7 ± 4.4* |
|  | *20 μM* | *62.5 ± 6.4* |
|  | *30 μM* | *59.3 ± 4.9* |
|  | *40 μM* | *34.0 ± 6.8* |
|  | *50 μM* | *22.6 ± 7.2* |
|  | *100 μM* | *11.1 ± 1.9* |
|  | *200 μM* | *4.8 ± 1.3* |
| ***^177^Lu-PSMA617*** | *0.01 μCi* | *97.1 ± 4.1* |
|  | *0.05 μCi* | *67.5 ± 1.5* |
|  | *0.1 μCi* | *59.7 ± 1.9* |
|  | *0.25 μCi* | *45.0 ± 2.6* |
|  | *0.5 μCi* | *38.3 ± 1.5* |

| ***^177^Lu-PSMA617 (μCi)/*** ***Cell Viability (% Mean ± SD)*** | ***+5 μM Genistein*** | ***+10 μM Genistein*** | ***+15 μM Genistein*** | ***+5 μM Baicalein*** | ***+10 μM Baicalein*** | ***+15 μM Baicalein*** |
| --- | --- | --- | --- | --- | --- | --- |
| *0.01* | *66.8 ± 1.9* | *59.4 ± 2.1* | *57.7 ± 1.5* | *80.1 ± 2.8* | *72.6 ± 2.7* | *68.8 ± 1.7* |
| *0.05* | *59.9 ± 2.1* | *44.9 ± 4.1* | *49.3 ± 0.8* | *65.3 ± 1.6* | *49.6 ± 2.9* | *46.6 ± 1.5* |
| *0.1* | *55.3 ± 1.3* | *37.9 ± 1.9* | *42.5 ± 0.8* | *55.5 ± 1.6* | *43.9 ± 2.9* | *43.7 ± 1.5* |
| *0.25* | *43.1 ± 1.9* | *32.1 ± 1.2* | *33.0 ± 0.5* | *41.2 ± 3.0* | *38.6 ± 0.9* | *37.8 ± 0.7* |
| *0.5* | *34.6 ± 0.2* | *23.9 ± 2.4* | *26.2 ± 0.2* | *33.3 ± 0.8* | *30.0 ± 3.0* | *31.6 ± 1.9* |

- ***Table S4.****Combined analysis of oxidative stress, DNA damage, and apoptosis induction in LNCaP cells.Reactive oxygen species (ROS) generation, DNA double-strand breaks (γ-H2AX foci), and Caspase-3 activity were measured after 24 h treatment. Data are presented as Relative Fluorescence Units (RFU, Mean ± SD) from three to five independent replicates (n=3–5).*

| *Treatment* | ***ROS (RFU)*** | ***γ-H2AX (RFU)*** | ***Caspase-3 (RFU)*** |
| --- | --- | --- | --- |
| *Control* | *8611 ± 231* | *1496 ± 27* | *9761 ± 85* |
| *^177^Lu-PSMA617* | *9345 ± 55* | *1594 ± 29* | *10218 ± 96* |
| *Genistein* | *9555 ± 83* | *1586 ± 22* | *9726 ± 61* |
| *Baicalein* | *8787 ± 129* | *1550 ± 30* | *9782 ± 72* |
| *^177^Lu-PSMA617 + Genistein* | *10579 ± 693* | *1725 ± 36* | *12261 ± 1057* |
| *^177^Lu-PSMA617 + Baicalein* | *10156 ± 175* | *1746 ± 38* | *11877 ± 721* |

- ***Table S5.****Membrane binding kinetics of ^177^Lu-PSMA617 in LNCaP cells.Binding rate (%) was measured at the indicated time points after pretreatment with flavonoids (10 μM, 24 h) followed by exposure to ^177^Lu-PSMA617. Data are presented as mean ± standard deviation (SD) from three independent replicates per time point.*

| *Time Point* | ***^177^Lu‑PSMA617 (Mean ± SD)*** | ***^177^Lu‑PSMA617 + Genistein (Mean ± SD)*** | ***^177^Lu‑PSMA617+ Baicalein (Mean ± SD)*** |
| --- | --- | --- | --- |
| *30 min* | *49.00 ± 1.11* | *49.40 ± 1.49* | *46.60 ± 1.63* |
| *60 min* | *49.71 ± 0.95* | *51.22 ± 2.50* | *53.00 ± 3.26* |
| *90 min* | *52.11 ± 3.10* | *56.74 ± 1.36* | *56.96 ± 2.88* |
| *120 min* | *57.09 ± 0.85* | *60.17 ± 1.17* | *59.25 ± 1.01* |

- ***Table S6.****Biodistribution of ^177^Lu-PSMA617 in LNCaP tumor-bearing mice. Radioactivity uptake expressed as percentage of injected dose per gram of tissue (%ID/g, Mean ± SD) at indicated time points post intravenous injection. Data are from three mice per time point (n=3).*

| *Tissue* | ***12 h*** | ***36 h*** | ***72 h*** | ***144 h*** |
| --- | --- | --- | --- | --- |
| *Blood* | *0.17 ± 0.05* | *0.33 ± 0.25* | *0.18 ± 0.01* | *0.24 ± 0.01* |
| *Tumor* | *5.51 ± 0.83* | *6.96 ± 1.24* | *4.21 ± 2.43* | *1.91 ± 0.52* |
| *Heart* | *0.07 ± 0.01* | *0.19 ± 0.10* | *0.07 ± 0.02* | *0.07 ± 0.00* |
| *Liver* | *0.08 ± 0.02* | *0.30 ± 0.05* | *0.11 ± 0.00* | *0.08 ± 0.02* |
| *Spleen* | *0.23 ± 0.06* | *0.53 ± 0.10* | *0.39 ± 0.07* | *0.53 ± 0.10* |
| *Lung* | *0.20 ± 0.12* | *0.28 ± 0.12* | *0.24 ± 0.05* | *0.21 ± 0.00* |
| *Kidney* | *0.65 ± 0.12* | *0.84 ± 0.06* | *0.49 ± 0.21* | *0.31 ± 0.02* |
| *Intestines* | *0.14 ± 0.02* | *0.26 ± 0.13* | *0.12 ± 0.02* | *0.17 ± 0.02* |
| *Brain* | *0.05 ± 0.01* | *0.12 ± 0.03* | *0.03 ± 0.01* | *0.04 ± 0.00* |
| *Bone* | *0.45 ± 0.04* | *0.32 ± 0.09* | *0.49 ± 0.18* | *0.51 ± 0.06* |
| *Muscle* | *0.13 ± 0.04* | *0.25 ± 0.01* | *0.19 ± 0.03* | *0.14 ± 0.04* |
| *Testis* | *0.09 ± 0.01* | *0.22 ± 0.03* | *0.13 ± 0.03* | *0.30 ± 0.01* |

***Table S6.A Longitudinal tumor volumes in LNCaP xenograft-bearing mice.****Tumor volume (mm^3^) was measured daily and calculated as V = 0.5 × length × width^2^. Data are presented as Mean ± Standard Error of the Mean (SEM) from five biological replicates (mice) per group (n=5).*

| ***Day*** | ***^177^Lu‑PSMA617 + Genistein*** | ***^177^Lu‑PSMA617 + Baicalein*** | ***^177^Lu‑PSMA617*** | ***Control*** | ***Genistein*** | ***Baicalein*** |
| --- | --- | --- | --- | --- | --- | --- |
| ***1*** | *201.5 ± 12.4* | *213.2 ± 15.3* | *198.9 ± 2.9* | *234.4 ± 24.7* | *249.0 ± 13.9* | *261.1 ± 9.4* |
| ***2*** | *224.7 ± 22.2* | *162.8 ± 17.7* | *256.4 ± 24.2* | *305.5 ± 37.9* | *306.1 ± 37.5* | *270.0 ± 18.7* |
| ***3*** | *165.9 ± 19.8* | *198.6 ± 13.5* | *217.0 ± 34.2* | *509.3 ± 59.6* | *421.7 ± 79.9* | *376.7 ± 49.1* |
| ***4*** | *216.0 ± 27.5* | *235.2 ± 20.9* | *188.7 ± 13.3* | *582.3 ± 42.6* | *391.5 ± 80.6* | *455.6 ± 29.5* |
| *5* | *168.9 ± 22.1* | *200.2 ± 13.9* | *156.7 ± 6.2* | *636.0 ± 89.4* | *625.1 ± 101.8* | *481.9 ± 47.0* |
| *6* | *138.5 ± 18.1* | *183.3 ± 13.5* | *244.5 ± 11.8* | *742.1 ± 78.9* | *558.7 ± 100.1* | *596.0 ± 41.5* |
| *7* | *126.0 ± 15.9* | *177.8 ± 11.2* | *290.5 ± 33.0* | *769.8 ± 94.8* | *742.7 ± 92.2* | *744.3 ± 51.3* |
| *8* | *102.7 ± 27.4* | *212.4 ± 12.9* | *299.6 ± 53.7* | *832.9 ± 93.8* | *748.0 ± 144.0* | *819.9 ± 85.0* |
| *9* | *89.7 ± 33.3* | *233.6 ± 21.0* | *338.8 ± 70.8* | *874.7 ± 111.5* | *755.4 ± 164.1* | *865.9 ± 87.1* |
| *10* | *90.2 ± 29.9* | *236.1 ± 20.7* | *381.8 ± 76.9* | *974.2 ± 133.2* | *833.1 ± 198.7* | *913.5 ± 92.0* |
| *11* | *90.9 ± 30.1* | *207.3 ± 10.8* | *415.8 ± 54.3* | *1026.5 ± 167.2* | *1003.8 ± 201.6* | *931.9 ± 84.6* |
| *12* | *82.3 ± 30.8* | *242.8 ± 1.3* | *406.1 ± 59.3* | *1123.4 ± 161.4* | *1073.5 ± 211.3* | *1012.6 ± 89.2* |
| *13* | *64.8 ± 34.4* | *276.5 ± 8.8* | *589.4 ± 79.5* | *1192.9 ± 216.0* | *1166.7 ± 283.1* | *1141.7 ± 148.6* |
| *14* | *81.5 ± 33.7* | *283.1 ± 13.4* | *720.4 ± 98.6* | *1299.4 ± 236.6* | *1208.7 ± 223.4* | *1197.9 ± 134.3* |
| *15* | *81.7 ± 35.0* | *310.1 ± 19.2* | *832.1 ± 89.2* | *1364.0 ± 271.6* | *1263.1 ± 265.0* | *1270.6 ± 126.9* |
| *16* | *92.9 ± 34.3* | *341.3 ± 16.5* | *920.6 ± 93.9* | *1402.4 ± 260.2* | *1360.5 ± 240.9* | *1323.9 ± 137.6* |
| *17* | *87.3 ± 34.4* | *336.1 ± 24.4* | *987.8 ± 100.6* | *1509.2 ± 286.4* | *1382.7 ± 264.9* | *1421.0 ± 111.1* |

***Table S6.B Daily Bliss synergy index (ΔE) analysis of combination therapies.***

*The Bliss Independence Model was used to quantify drug interactions. ΔE = E_observed – E_expected. For each day, the ΔE was calculated based on the mean tumor growth inhibition of each group. The standard error of the mean (SEM) for ΔE was propagated from the SEMs of the observed and expected effects.*

| ***Day*** | ***^177^Lu‑PSMA617 + Genistein (Mean ΔE ± SEM)*** | ***^177^Lu‑PSMA617 + Baicalein (Mean ΔE ± SEM)*** |
| --- | --- | --- |
| *2* | *0.032 ± 0.030* | *-0.107 ± 0.023* |
| *3* | *0.138 ± 0.038* | *0.076 ± 0.018* |
| *4* | *0.166 ± 0.028* | *0.068 ± 0.015* |
| *5* | *0.278 ± 0.032* | *0.109 ± 0.017* |
| *6* | *0.379 ± 0.036* | *0.157 ± 0.019* |
| *7* | *0.436 ± 0.039* | *0.180 ± 0.020* |
| *8* | *0.478 ± 0.041* | *0.191 ± 0.021* |
| *9* | *0.510 ± 0.043* | *0.195 ± 0.022* |
| *10* | *0.535 ± 0.044* | *0.195 ± 0.022* |
| *11* | *0.553 ± 0.045* | *0.192 ± 0.022* |
| *12* | *0.567 ± 0.046* | *0.185 ± 0.023* |
| *13* | *0.576 ± 0.047* | *0.176 ± 0.023* |
| *14* | *0.581 ± 0.047* | *0.164 ± 0.023* |
| *15* | *0.583 ± 0.047* | *0.151 ± 0.023* |
| *16* | *0.582 ± 0.047* | *0.136 ± 0.023* |
| *17* | *0.578 ± 0.047* | *0.121 ± 0.023* |

***Table S7. Quantitative histopathological analysis of tumor tissues. Data are presented as mean ± standard deviation (SD), n = 3 per group.***

| *Treatment Group* | *Necrotic cell ratio（%）* | *Cellular fibrosis（%）* | *Inflammatory Cell Infiltration（40X）* | *Apoptosis count（40X）* |
| --- | --- | --- | --- | --- |
| ***^177^Lu‑PSMA617*** | *1.33 ± 0.58* | *7.00 ± 1.73* | *4.00 ± 1.73* | *1.00 ± 0.00* |
| ***^177^Lu‑PSMA617 + Genistein*** | *17.33 ± 4.62* | *9.33 ± 1.15* | *15.67 ± 0.58* | *3.67 ± 0.58* |
| ***^177^Lu‑PSMA617 + Baicalein*** | *6.67 ± 1.15* | *3.67 ± 1.15* | *4.67 ± 1.15* | *1.33 ± 0.58* |
| ***Genistein*** | *1.33 ± 1.15* | *7.00 ± 1.73* | *4.00 ± 1.73* | *1.00 ± 0.00* |
| ***Baicalein*** | *0.67 ± 0.58* | *0.33 ± 0.58* | *2.00 ± 0.00* | *1.00 ± 0.00* |
| ***Control*** | *0.33 ± 0.58* | *0.33 ± 0.58* | *2.00 ± 1.00* | *0.33 ± 0.58* |

***Data Availability***

*Raw RNA-seq data will be deposited in the Gene Expression Omnibus (GEO) under accession number****GSEXXXXXX****(to be provided upon acceptance).*

*All other data supporting the findings of this study are available from the corresponding authors upon reasonable request.*
